# The complete mitochondrial genome of the tapeworm *Cladotaenia vulturi* (Cestoda: Paruterinidae): gene arrangement and phylogenetic relationships with other cestodes

**DOI:** 10.1186/s13071-016-1769-x

**Published:** 2016-08-31

**Authors:** Aijiang Guo

**Affiliations:** 1 State Key Laboratory of Veterinary Etiological Biology, Key Laboratory of Veterinary Parasitology of Gansu Province, Lanzhou Veterinary Research Institute, Chinese Academy of Agricultural Sciences, Lanzhou, 730046 Gansu Province People’s Republic of China; 2 Jiangsu Co-Innovation Center for Prevention and Control of Important Animal Infectious Diseases and Zoonoses, Yangzhou, 225009 Jiangsu Province People’s Republic of China

**Keywords:** Mitochondrial genome, *Cladotaenia vulturi*, Comparative genomics, Phylogeny, Cestoda, Cyclophyllidea

## Abstract

**Background:**

Tapeworms *Cladotaenia* spp. are among the most important wildlife pathogens in birds of prey. The genus *Cladotaenia* is placed in the family Paruterinidae based on morphological characteristics and hosts. However, limited molecular information is available for studying the phylogenetic position of this genus in relation to other cestodes.

**Methods:**

In this study, the complete mitochondrial (mt) genome of *Cladotaenia vulturi* was amplified using “Long-PCR” and then sequenced by primer walking. Sequence annotation and gene identification were performed by comparison with published flatworm mt genomes. The phylogenetic relationships of *C. vulturi* with other cestode species were established using the concatenated amino acid sequences of 12 protein-coding genes with Bayesian Inference and Maximum Likelihood methods.

**Results:**

The complete mitochondrial genome of the *Cladotaenia vulturi* is 13,411 kb in size and contains 36 genes. The gene arrangement of *C. vulturi* is identical to those in *Anoplocephala* spp. (Anoplocephalidae), *Hymenolepis* spp. (Hymenolepididae) and *Dipylidium caninum* (Dipylidiidae), but different from that in taeniids owing to the order shift between the tRNA (L1) and tRNA (S2) genes. Phylogenetic analyses based on the amino acid sequences of the concatenated 12 protein-coding genes showed that the species in the Taeniidae form a group and *C. vulturi* is a sister taxon to the species of the family Taeniidae.

**Conclusions:**

To our knowledge, the present study provides the first molecular data to support the early proposal from morphological evidence that the Taeniidae is a sister group to the family Paruterinidae. This novel mt genome sequence will be useful for further investigations into the population genetics, phylogenetics and systematics of the family Paruterinidae and inferring phylogenetic relationships among several lineages within the order Cyclophyllidea.

**Electronic supplementary material:**

The online version of this article (doi:10.1186/s13071-016-1769-x) contains supplementary material, which is available to authorized users.

## Background

Tapeworms *Cladotaenia* spp. are among the most important wildlife pathogens of birds of prey. Historically, the genus *Cladotaenia* Cohn, 1901 was created within Taeniidae based on morphological similarities [1]. The uterus of *Cladotaenia* spp. consists of median stem and lateral branches, similar to the species in the family Taeniidae [1]. While the life-cycles of all species in the family Taeniidae involve two mammals, the definitive hosts of the genus *Cladotaenia* are birds of prey [1]. Thus, authorities have removed the genus *Cladotaenia* from the family Taeniidae to the Paruterinidae [1] based on morphological and biological grounds. In the current classification, the family Paruterinidae includes all of the cyclophyllidean cestodes with paruterine organs which cannot be related to other families having similar uterine structures [1]. Five genera (*Paruterina*, *Cladotaenia*, *Culcitella*, *Laterotaenia* and *Matabelea*) are considered as the Paruterinidae [1]. The systematics of the Paruterinidae have been problematic [2] and there are few studies investigating the phylogeny of the Paruterinidae including *Cladotaenia* [2, 3].

Molecular tools have been used to study phylogenetic relationships among groups of cyclophyllidean tapeworms [4–9]. However, the limited availability of sequence data has greatly impeded studies of the phylogenetic relationships among families in the order Cyclophyllidea. Compared to some genes (*18S*, *12S* and *cox1*), complete mtDNA sequences are more informative at the generic level [8, 10]. Complete mitochondrial DNA sequences can provide not only individual and combined mt genes but also gene order. Mitochondrial gene rearrangement is generally a rare event occurring over long periods of evolutionary time [11], although mitochondrial sequences evolve rapidly in metazoans [12]. Relative gene rearrangement found in a group of species is considered evidence of having closer relationship [13]. Indeed, gene rearrangement comparisons used as phylogenetic tools have been well discussed [14, 15] and used to analyze several evolutionary relationships in echinoderms [16], arthropods [17, 18], gastropods [19] and annelids [20]. However, only 37 complete cestode mitochondrial genome sequences are available for species of the order Cyclophyllidea in the NCBI database and no complete mitochondrial genome was available in the family Paruterinidae until now.

The present study aimed to (i) characterize the complete mt genome of *C. vulturi*, the first representative of the family Paruterinidae; (ii) compare its mt gene content, arrangement with those of other cestodes; and (iii) infer the phylogenetic position of *C. vulturi* in relation to other tapeworms based on the concatenated mt amino acid sequences.

## Methods

### Sample collection

In 2013, an emaciated wild *Aquila nipalensis* (steppe eagle) was found in Kashi, Xinjiang, China, and then was taken to a Zoo where it died soon after. Six tapeworms were found in the intestine of this dead *A. nipalensis* with scolices firmly attached to the intestinal wall. In order to obtain the whole tapeworms, intestinal segment with the attached scolices was excised and immersed in water for 5–6 h for separating and stretching the tapeworms. Each of the six tapeworms was morphologically identified by light microscopy and electron microscopy. Individual scolices were fixed in 2.5 % gultaraldehyde. The structure of the scolex was observed by scanning electron microscope. Mature and gravid proglottides were placed in 70 % ethanol solution and stained with hematoxylin and eosin staining kit (Beijing CellChip Biotechnology Co. Ltd, Beijing, China). Morphological observation included rostellum, hooks, suckers and testes and ovary [1]. All six tapeworms were identified as *C. vulturi* according to keys to the cestode parasites of vertebrates [1]. The tapeworm used for DNA extraction was kept in 70 % ethanol solution and two tapeworms were fixed in 5 % formalin solution; these were archived in the Parasitological Museum of Lanzhou Veterinary Research Institute, Lanzhou, Gansu, China, under collection number PML 1996.

### DNA extraction, amplification and sequencing

Total genomic DNA was extracted from a single adult using the Tissue DNA kit (OMEGA, Doraville, USA) according to the manufacturer’s protocol. First, the partial gene fragments for *nad1*, *nad5* and *rrnS* were amplified using published primer sets designed according to the published conserved regions of the cestode species [21, 22] (Additional file 1: Table S1). PCR reactions for these three partial fragments were performed in a 50 μl reaction volume consisting 25 μl of LA Taq Premix buffer (TaKaRa Biotechnology Co, Dalian, China), 22.5 μl of sterile deionized water, 0.5 μl of each primer (50 pmol/μl), and 1.5 μl of DNA template (40 ng/μl) with the following conditions: 94 °C for 5 min, then 94 °C for 10 s, 55 °C for 30 s, 72 °C for 1 min for 30 cycles, followed by 72 °C for 7 min. Then the three overlapping long PCR fragments were amplified using Long-PCR primer sets, designed according to the above mentioned three primer sets (Additional file 1: Table S1). PCR reactions were conducted in 50 μl mixture including 25 μl of LA Taq Premix buffer (TaKaRa Biotechnology Co, Dalian, China), 22.5 μl of sterile deionized water, 0.5 μl of each primer (50 pmol/μl), and 1.5 μl of DNA template (40 ng/μl). The amplification conditions of the three long-PCR reactions consisted of 94 °C for 5 min, then 94 °C for 10 s, 50 °C for 30 s, 68 °C for 10 min for 30 cycles, followed by 68 °C for 10 min. PCR products of the *nad1*, *nad5* and *rrnS* gene fragments were purified, cloned and sequenced. PCR products of the three Long-PCR fragments were sequenced in both directions using the ‘primer walking’ method after gel purification. The complete mtDNA sequence was assembled using Sequencher ver. 3.1.1 (GeneCodes) by overlapping regions between the long PCR fragments and three partial sequences.

### Sequence annotation and gene identification

MacVector 8.1.2 was used to annotate the sequence through comparison with published flatworm genomes. The reading frames were translated using flatworm mt code (translation table 9). Twelve mt protein-coding genes were determined by similarity comparisons of inferred amino acid sequences to those of other flatworm mtDNAs. Two ribosomal RNA genes were identified by finding gene boundaries based on comparison with other flatworm mtDNA sequences. The 22 tRNA genes were searched by using the tRNAscan-SE software and by eye detecting potential secondary structures and anticodon sequences. Secondary structures in the two larger non-coding regions were predicted with the Mfold program [23].

### Phylogenetic analysis

A total of 45 mt genome sequences were used in the phylogenetic analysis, including all of the mitochondrial genomes of cyclophylidean tapeworms available on GenBank [10], the mt genome for *C. vulturi* identified in this study, six mt genomes of species of the Pseudophyllidea, and the mt genome of *Schistosoma japonicum* (Trematoda) as the outgroup. Phylogenetic analyses were performed using the concatenated amino acid sequences of 12 protein-coding genes. The amino acid sequences for each protein-coding gene of the 45 species were aligned individually using MAFFT 7.122 [24]. Poorly aligned positions were discarded by Gblocks [25], using the option for a less stringent selection. A single alignment for phylogenetic analyses was conducted by concatenating all amino acid alignments of the 12 protein-coding genes. The optimal model for phylogenetic analysis was selected by ProtTest [26]. The phylogenetic trees were constructed using Maximum Likelihood (ML) and Bayesian Inference (BI) analyses. ML analysis was conducted by PhyML 3.0 software [27] with MtArt + I + G model, and 100 bootstrap replicates were chosen to calculate bootstrap support for ML trees. BI analysis was performed using MrBayes 3.2 software [28] with mtZoa model (rates = gamma, ngammacat = 5), as suggested by Rota-Stabelli et al. [29]. Two chains were run for 5,000,000 generations and sampled every 1,000 generations. The first 25 % of the trees were treated as 'burn-in' and Bayesian posterior probabilities were calculated for the remaining trees.

### Relative synonymous codon usage

To examine the codon usage variation among the 12 protein-coding mt gene sequences of cestode species, relative synonymous codon usage values (RSCU) of different codons [30] in each representative cestode species were calculated using CodonW (Version 1.4.2). Then a dendrogram was produced by the hierarchical clustering algorithm implemented in Gene Cluster (V3.0) based on the RSCU values.

## Results and discussion

### Amplification of the mtDNA of *Cladotaenia vulturi*

**Fig. 1:**
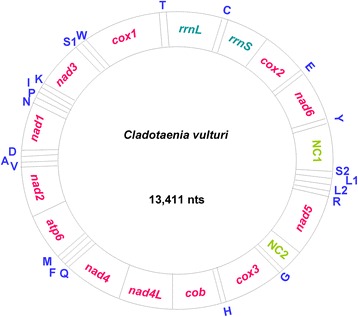
The mitochondrial genome of *Cladotaenia vulturi*. Genome organization of the complete mitochondrial genome of *Cladotaenia vulturi* is a circular DNA molecule containing 36 genes. All genes are coded by the same DNA strand and are transcribed in the same direction. Protein-coding and rRNA genes are shown with the standard nomenclature. tRNA genes are designated with the one letter code of their corresponding amino acids, where L_1_ and L_2_ represent individual leucine codons CUN and UUR; and S_1_ and S_2_ represent serine codons AGN and UCN, respectively. “NC” refers to the non-coding region

The three long fragments with three overlapping fragments of *nad1*, *nad5* and *rrnS* jointly represent the complete mt genome of *C. vulturi*. The assembled sequence shows that the mt genome of *C. vulturi* (GenBank accession No. KU559932) is circular, 13,411 bp long (Fig. 1), making it the smallest among the mt genomes of cestode species reported to date (range 13,482–14,459 bp). The nucleotide composition of AT in mtDNA of *C. vulturi* is 74.64 %, which is slightly higher than that reported for other cestode species to date.

### Gene content and organization

Thirty-six genes including 12 protein-coding genes (*nad1-6*, *nad4L*, *cox1-3*, *cob* and *atp6*), two ribosomal RNA genes (*rrnS* and *rrnL*), and 22 transfer RNA (*trn*) genes were identified in the mt genome of *C. vulturi* (Table 1). As with previously reported flatworm mt genomes, *C. vulturi* lacks the *atp8* gene, which is found in animal mt DNAs. All of these genes were coded unidirectionally, on the plus strand (Fig. 1), a condition observed in other flatworms. The gene arrangement of *C. vulturi*, however, exhibited a slight difference from those of species in the family Taeniidae, with the reversal in the position of *trnS* (UCA) and *trnL* (CUA).

**Table 1:** Summary data for the annotated mitochondrial genome of *Cladotaenia vulturi*

Gene	Position/ length	Initiation/ termination codon	Anticodon	No. of amino acids	No. of intergenic nucleotides^a^
*cox1*	1–1,584/1,584	GTG/TAG	–	527	+6
*trnT*	1,575–1,635/61	–	TGT	–	-10
*rrnL*	1,636–2,599/964	–	–	–	0
*trnC*	2,600–2,662/63	–	GCA	–	0
*rrnS*	2,663–3,377/715	–	–	–	0
*cox*2	3,378–3,959/582	GTG/TAA	–	193	0
*trnE*	3,962–4,027/66	–	TTC	–	+2
*nad*6	4,030–4,485/456	ATG/TAG	–	151	+2
*trnY*	4,489–4,552/64	–	GTA	–	+3
Non-coding region (NC1)	4,553–4,687/135	–	–	–	0
*trnS2*	4,688–4,750/63	–	TGA	–	0
*trnL1*	4,759–4,823/65	–	TAG	–	+8
*trnL2*	4,832–4,894/63	–	TAA	–	+8
*trnR*	4,902–4,958/57	–	ACG	–	+7
*nad*5	4,960–6,528/1569	ATG/TAA	–	522	+1
Non-coding region (NC2)	6,529–6,636/108	–	–	–	0
*trnG*	6,637–6,705/69	–	TCC	–	0
*cox3*	6,707–7,351/645	GTG/TAA	–	214	+1
*trnH*	7,355–7,420/66	–	GTG	–	+3
*cytb*	7,424–8,517/1094	ATG/TA	–	364	+3
*nad4L*	8,518–8,778/261	ATG/TAA	–	86	0
*nad4*	8,739–9,983/1245	GTG/TAA	–	414	-40
*trnQ*	9,984–10,046/63	–	TTG	–	0
*trnF*	10,047–10,108/62	–	GAA	–	0
*trnM*	10,105–10,167/63	–	CAT	–	-4
*atp6*	10,169–10,684/516	ATG/TAA	–	171	+1
*nad2*	10,689–11,564/876	ATG/TAA	–	291	+4
*trnV*	11,574–11,637/64	–	TAC	–	+9
*trnA*	11,643–11,707/65	–	TGC	–	+5
*trnD*	11,711–11,776/66	–	GTC	–	+3
*nad1*	11,779–12,669/891	ATG/TAA	–	296	+2
*trnN*	12,673–12,734/62	–		–	+3
*trnP*	12,736–12,799/64	–		–	+1
*trnI*	12,800–12,862/63	–		–	0
*trnK*	12,868–12,930/63	–		–	+5
*nad3*	12,934–13,281/348	GTG/TAG	–	115	+3
*trnS1*	13,280–13,338/59	–	GCT	–	-2
*trnW*	13,343–13,405/63	–	TCA	–	+4

^a^Minus sign (-) and plus sign (+) show the length of overlap and intergenic gap between two adjacent genes respectively

### Initiation and termination codons and protein-coding genes

Initiation and termination codons inferred from each protein-coding gene are shown in Table 1. The inferred start codon for seven of the 12 protein-coding genes in *C. vulturi* was ATG, while the inferred start codon for the other five protein-coding genes (*cox1-3*, *nad3* and *nad4*) was GTG. For the *nad4* gene, there was another eligible start codon (ATG) in-frame that is a reasonable alternative, and the sizes of the inferred amino terminal residues from the start codons GTG and ATG were 414 aa and 412 aa, respectively. Eleven of the 12 protein-coding genes in the mt genome of *C. vulturi* can be inferred to end with a complete stop codon (three are TAG and eight are TAA). Only one abbreviated stop codon (TA) was found, for the *cytb* gene; abbreviated stop codons (T or TA) have also been found in the mt protein-coding genes of other cestode species [31]. An alternative complete stop codon (TAG) for the *cytb* gene is possible in-frame that would share one nt (G) with the downstream *nad4L* gene. The length for each of 12 proteins, deduced form each protein-coding gene, was similar to those already characterized for other cestodes (Table 2).

**Table 2:** Properties of protein-coding genes, length of the mt genomes and rRNA genes and AT content of cestode mt genomes

Gene	Species											
	*T. s.*	*T. a.*	*E. g.*	*E. m.*	*H. k.*	*H. p.*	*V. m.*	*D. c.*	*A. p.*	*A. m.*	*H. d.*	*C. v.*
Number of aa												
*cox3*	214	214	215	215	214	214	215	215	214	214	216	214
*cytb*	355	355	355	355	355	354	355	360	366	366	365	364
*nad4L*	86	86	86	86	86	86	86	86	86	86	86	86
*nad4*	403	417	419	404	416	416	419	415	415	415	409	414
*atp6*	171	171	170	171	172	171	171	171	171	171	171	171
*nad2*	293	293	293	293	295	294	291	290	291	291	293	291
*nad1*	297	297	297	297	297	298	297	297	296	296	296	296
*nad3*	115	115	115	115	113	113	115	114	115	115	115	115
*cox1*	539	539	557	535	543	538	539	580	530	529	517	527
*cox2*	193	191	193	193	194	194	192	191	191	191	192	193
*nad6*	150	150	151	151	150	149	152	151	152	152	152	151
*nad5*	522	522	523	524	522	524	522	521	526	526	524	522
Length of rRNA gene (bp)												
rrnL	980	975	967	983	960	961	963	970	981	973	967	964
rrns	705	731	726	704	728	730	727	729	724	724	709	715
Deduced initiation codon												
*cox3*	ATG	ATG	ATG	ATG	ATG	ATG	ATG	ATG	ATG	ATG	ATG	GTG
*cytb*	ATG	ATG	ATG	ATG	ATG	ATG	ATG	ATG	GTG	GTG	ATG	ATG
*nad4L*	ATG	ATG	GTG	GTG	ATG	ATG	ATG	ATG	ATG	GTG	ATG	ATG
*nad4*	GTG	ATG	ATG	ATG	GTG	ATG	ATG	GTG	ATG	GTG	ATT	GTG
*atp6*	ATG	ATG	ATG	ATG	ATG	ATG	ATG	ATG	ATG	ATG	ATG	ATG
*nad2*	ATG	ATG	ATG	ATG	ATG	ATG	ATG	ATG	ATG	ATG	ATG	ATG
*nad1*	ATG	ATG	GTG	ATG	GTG	ATG	ATG	ATG	ATG	GTG	ATG	ATG
*nad3*	ATG	ATG	ATG	ATG	ATG	ATG	ATG	ATG	ATG	ATG	ATG	GTG
*cox1*	ATG	ATG	ATG	ATG	ATG	ATG	ATG	ATG	ATG	ATG	TTT	GTG
*cox2*	ATG	ATG	GTG	GTG	ATG	ATG	ATG	ATG	ATG	ATG	ATG	GTG
*nad6*	ATG	GTG	ATG	ATG	GTG	ATG	ATG	ATG	ATG	ATG	ATG	ATG
*nad5*	ATG	ATG	ATG	ATG	ATG	ATG	ATG	ATG	ATG	ATG	ATG	ATG
Deduced termination codon												
*cox3*	TAG	TAG	TAG	TAG	TAA	TAA	TAG	T	TA	TA	TAG	TAA
*cytb*	TAA	TAA	TAA	TAA	TAA	TAA	TAA	TAG	TAG	TAA	TAG	TA
*nad4L*	TAA	TAG	TAA	TAG	TAG	TAA	TAG	TAG	TAG	TAG	TAG	TAA
*nad4*	TAG	TAG	TAG	TAG	TAG	TAA	TAA	TAA	TAA	TAG	TAG	TAA
*atp6*	TAA	TAA	TAG	TAG	TAG	TAG	TAA	TAG	TAG	TAG	TAG	TAA
*nad2*	TAA	TAA	TAG	TAG	TAA	TAA	TAG	T	TAG	TAG	TAG	TAA
*nad1*	T	T	TAA	TAG	TAG	TAG	TAG	TAA	TAA	TAA	TAG	TAA
*nad3*	TAG	TAG	TAG	TAA	T	TAG	TAG	TAG	TAA	TAA	TAG	TAG
*cox1*	TAG	TAA	TAA	TAG	TAA	TAG	TAG	TAA	TAA	TAG	T	TAG
*cox2*	TAG	TAG	TAG	TAG	T	T	TAG	TAG	TAG	TAG	TAA	TAA
*nad6*	TAG	TAA	TAG	TAA	TAG	TAA	TAA	TAG	TAG	TAG	TAA	TAG
*nad5*	TAA	TAA	TAA	TAA	TAG	TAA	TAA	TAA	TAG	TAA	TAG	TAA
Length of mt genome (bp)												
–	13,709	13,703	13,610	13,738	13,792	13,482	13,582	14,296	14,459	13,759	13,900	13,411
AT content of complete mt genome (%)												
–	72.05	71.40	67.02	69.04	72.77	71.40	71.37	72.89	71.05	70.80	71.04	74.64

Note: Results obtained from GenBank accessions

*Abrreviations*: *T. s. Taenia solium*, *T. a. Taenia asiatica*, *E. g. Echinococcus granulosus*, *E. m. Echinococcus multilocularis*, *H. k. Hydatigera krepkogorski*, *H. p. Hydatigera parva*, *V. m. Versteria mustelae*, *D. c. Dipylidium caninum*, *A. p. Anoplocephala perfoliata*, *H. d. Hymenolepis diminuta*, *D. n. Diphyllobothrium nihonkaiense*, *D. l. Diphyllobothrium latum*, *D. g. Diplogonoporus grandis*, *D. b. Diplogonoporus balaenopterae*, *S. e. Spirometra erinaceieuropaei*, *C. v. Cladotaenia vulturi*

Overlap was found between *nad4L* and *nad4*, as is common among metazoan mt genomes for these two genes [12]. Overlap also appeared between *trnF* and *trnM*. Indeed, overlaps between tRNA genes are common among mt genomes [12]. It is noteworthy that overlaps were detected between the *cox1* and *trnT* genes (10 bp overlap), and the *nad3* and *trnS1* (AGN) genes (2 bp overlap) in the mt genome of *C. vulturi*. However, the overlap between protein-coding and tRNA genes is uncommon. Among the tapeworms, an overlap between protein-coding and tRNA genes has been found only between the *nad1* and *trnD* genes in *Schistosoma haematobium* [32].

### Ribosomal and transfer RNA genes

The ribosomal RNA genes *rrnS* and *rrnL* of *C. vulturi* are 715 bp and 964 bp long, respectively (Table 1). *rrnS* is located between *trnC* and *cox2* and *rrnL* is positioned between *rrnT* and *rrnC*. The sizes and positions of *rrnS* and *rrnL* in *C. vulturi* are similar to those in other cestodes. Twenty-two transfer RNA genes (tRNA) were identified in *C. vulturi* with the length of the individual tRNA genes ranging between 57 and 69 bp (Table 1). Eighteen of the 22 tRNA nucleotide sequences have the potential to form a standard cloverleaf structure, the exceptions comprising *trnS1* (AGN), *trnS2* (UCN), *trnC* and *trnR*, all lacking a DHU arm (Additional file 2: Figure S1). Inferred tRNA secondary structures for *C. vulturi* exhibited no differences when compared to other cestode representatives.

### Non-coding regions

The two largest non-coding regions in *C. vulturi* are designated as NC1 and NC2 (Table 1; Fig. 1). NC1 is 135 bp in length and is located between *trnY* and *trnS2*. NC2 is 108 bp long and is positioned between *nad5* and *trnG*. These two large non-coding regions were predicted each to form two stem-loop structures (Additional file 3: Figure S2). One stem-loop structure in NC1 has ten nucleotide pairs and a loop of 3 nt while the other has 27 nucleotide pairs with a loop of 13 nt (Additional file 3: Figure S2a). In the case of NC2, one stem-loop structure has six nucleotide pairs and a loop of 5 nt and the other has 31 nucleotide pairs with a loop of 13 nt (Additional file 3: Figure S2b). The longer secondary structure of NC1 includes a T-rich region (Additional file 3: Figure S2a). Palindromes and several copies of the dinucleotide AT were found in the two non-coding regions. These potential secondary structures and unique features of AT or T richness and palindromes are also found in the non-coding regions of representatives of three other cestode genera, i.e. *Taenia*, *Echinococcus* and *Hymenolepis* [33]. The function of these secondary structures in non-coding regions remains unknown. However, similar secondary structures have been shown to initiate replication and transcription in mammals [32], and it is possible that the secondary structures in the non-coding regions of tapeworms have a similar function.

### Phylogenetic analysis

**Fig. 2:**
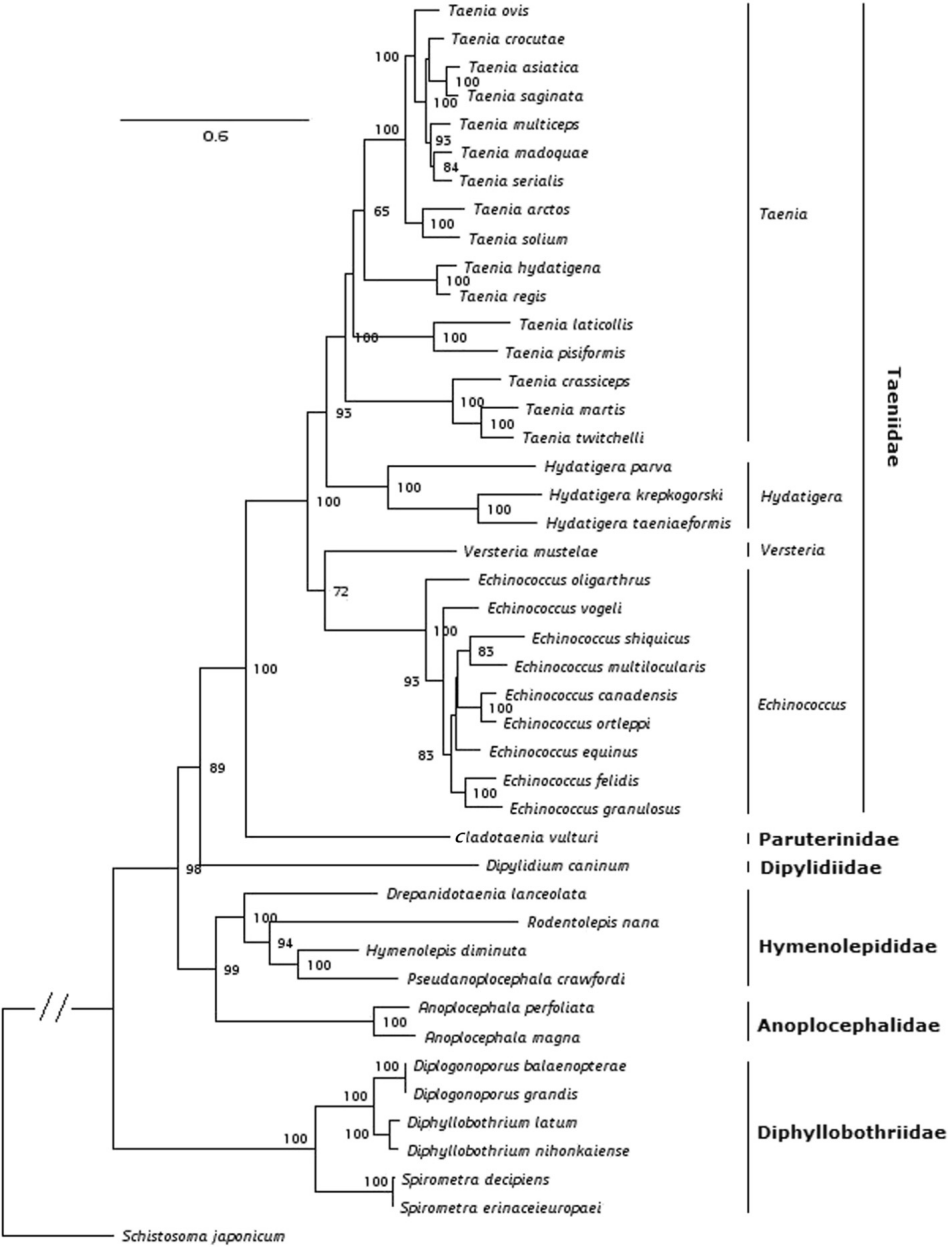
Phylogenetic relationships among members of the Anoplocephalidae, Dipylidiidae, Hymenolepididae, Paruterinidae and Taeniidae. Tree from a Maximum Likelihood analysis using deduced amino acids of 12 protein-coding genes with bootstrapping frequency values shown at the nodes

Bayesian inference and Maximum Likelihood methods were used to construct the phylogenetic trees; as these exhibited identical topology and support, only the topology resulting from the ML analysis is shown in Fig. 2. Representatives of the Pseudophyllidea (Diphyllobothriidae) and Cyclophyllidea (Anoplocephalidae, Dipylidiidae, Hymenolepididae, Paruterinidae and Taeniidae) formed strongly supported independent clades. The Taeniidae included four different clades and was resolved as an independent group within the order Cyclophyllidea (Fig. 2). The Hymenolepididae exhibited a sister-group relationship with the Anoplocephalidae, consistent with what has been reported in studies based on 18S rDNA [4, 9] and 12S rDNA [34]. This is also in accordance with cladistic analyses based on morphological characters by Hoberg et al. [3]. Representatives of the Taeniidae formed a strongly supported clade and *C. vulturi* appeared as the sister taxon with maximum support.

Gene rearrangement comparisons used as phylogenetic tools have been used to analyze several evolutionary relationships, the closest taxa sharing an identical gene order [16]. The mt genome of *C. vulturi* has a different gene order to those of species in the family Taeniidae. This result lends further support to the current classification, where the genus *Cladotaenia* is not a member of the family Taeniidae [1].

In the present phylogeny, four clades were formed by the genera *Taenia*, *Hydatigera*, *Versteria* and *Echinococcus* within the Taeniidae, coincident with previously published estimations of the relationships between the suprageneric taxa within the family and the Cyclophyllidea [10, 34]. Furthermore, the internal topologies of all of the clades within the Taeniidae are fully identical to those inferred from amino acids of the 12 protein-coding genes using *Dipylidium caninum* as the outgroup [10, 34]. Additionally, a sister-group relationship between *Echinococcus* spp. and *V. mustelae* was supported and identical to those inferred from both mt protein-coding genes and nuclear genes (*pepck* and *pold*) using *Dipylidium caninum* as the outgroup, but inconsistent to the phylogenies reconstructed using 18S rDNA suggesting that *V. mustelae* is a sister taxon to *Taenia* spp. [10, 34]. Additional evidence is required to fully understand the relationship between *Versteria* and *Echinococcus*. Furthermore, *C. vulturi* is closely related to the species in the family Taeniidae with strong support in the topology of the current analysis, further supporting the hypothesis of a sister-group relationship between the Paruterinidae and Taeniidae based on an early morphological study by Hoberg et al. [3].

### Relative synonymous codon usage

Analysis of the relative synonymous codon usage (RSCU) of the 12 protein-coding genes may shed light on the general codon usage patterns of evolution among the cestode species. Numbers of each amino acid and their RSCU values in the mt protein-coding genes of a range of the cestode species used to infer the phylogeny in Fig. 2 were calculated in this study (Additional file 4: Table S2). The result showed that the same codons tend to be most common in all species. That means the common pattern of bias is observed. The most common codons are UUA, ACU, GCU, UGA in every case, while UAA, UAG, CGC are the least common codons. The cluster analysis based on the divergence in codon usage (Additional file 5: Figure S3) showed that four groups were classified by RSCU with members of the Taeniidae dispersed in different clusters with taxonomic content different from the phylogenetic hypothesis based on the 12 protein-coding gene sequences. The distance tree inferred from the values of RSCU failed to recover the topology expected from the known systematic relationships among cestode species. This result supports the opinion that base composition differences may affect tree topologies [35, 36].

The mt genome of *C. vulturi* described here provides data for the first member of the family Paruterinidae sequenced. Additional molecular data with more taxa from a wide range of lineages, including *Cladotaenia* spp. and other species in the Paruterinidae, is required to assess the phylogenetic position of the family Paruterinidae within the order Cyclophyllidea.

## Conclusions

In the current study, the complete mt genome of *C. vulturi* was sequenced and characterized. It contains 36 genes, including 22 transfer RNA genes, two ribosomal RNA genes and 12 protein-coding genes. Phylogenetic analyses inferred from amino acid sequences of the 12 protein-coding genes suggest that *C. vulturi* is a sister taxon to the species of the family Taeniidae. The novel mt genome sequence provided here will be useful in further investigations of the comparative mitochondrial genomics and systematics of parasitic tapeworms.

## Additional files

Additional file 1:
**Table S1.** Primers used for amplifying mtDNA fragments and their positions in the mt genome of *Cladotaenia vulturi.* (DOC 30 kb) Additional file 2:
**Figure S1.** Deduced secondary structures for the four tRNA genes of *Cladotaenia vulturi* mt genome. Only these four tRNA genes lack typical cloverleaf structure. (DOC 168 kb) Additional file 3:
**Figure S2.** Putative secondary structures of the two non-coding regions in *Cladotaenia vulturi* mt genome. **a** The long non-coding region (NC1) located between *trnY* and *trnS2*. **b** The short non-coding region (NC2) between *nad5* and *trnG.* (DOC 102 kb) Additional file 4:
**Table S2.** Codon frequencies (N) and relative synonymous codon usage (RSCU) values in 12 mt protein-coding genes of cestode species. (DOC 1068 kb) Additional file 5:
**Figure S3.** Distance tree based on the RSCU values for 12 mt protein-coding genes of cestode species used in the phylogenetic analyses. (DOC 147 kb)

## Abbreviations

BI: Bayesian inference
ML: Maximum likelihood
Mt: Mitochondrial
