# Supplementary material for: The complete mitochondrial genome of the tapeworm Cladotaenia vulturi (Cestoda: Paruterinidae): gene arrangement and phylogenetic relationships with other cestodes
Source: Parasit Vectors. 2016 Aug 31;9(1):475. doi: 10.1186/s13071-016-1769-x (PMC5006517; doi:10.1186/s13071-016-1769-x)
Supplement: Additional file 1: — Table S1. Primers used for amplifying mtDNA fragments and their positions in the mt genome of Cladotaenia vulturi. (DOC 30 kb) [file 13071_2016_1769_MOESM1_ESM.doc]

***Additional file 1: Table S1. Primers used for amplifying mtDNA fragments and their position in the mt genome of Cladotaenia vulturi***

| **Primer (positions)** | **Sequence (5’ to 3’)** |
| --- | --- |
| AP1F_ND1 (12517–12542) | CARTTTCGTAAGGGBCCWAAWAAGGT |
| AP1R_rrnS (3362–3396) | AATTCATTTAAAGTTACCTTGTTACGACTTACCTC |
| AP2F_rrnS (2850–2880) | AGGGGATAGGRCACAGTGCCAGCATCTGCGG |
| AP2R_ND5 (5927–5949) | GGAAAHCTAGCACTCTTDGTAA |
| AP3F_ND5 (5295–5325) | TATATGAGTTAGTTTTAAGCATTAATTATGG |
| AP3R_ND1 (13361–13386) | CCATTTCYTGAAGTTAACAGCATCA |
