# Supplementary material for: The complete mitochondrial genome of the tapeworm Cladotaenia vulturi (Cestoda: Paruterinidae): gene arrangement and phylogenetic relationships with other cestodes
Source: Parasit Vectors. 2016 Aug 31;9(1):475. doi: 10.1186/s13071-016-1769-x (PMC5006517; doi:10.1186/s13071-016-1769-x)
Supplement: Additional file 2: — Figure S1. Deduced secondary structures for the four tRNA genes of Cladotaenia vulturi mt genome. Only these four tRNA genes lack typical cloverleaf structure. (DOC 168 kb) [file 13071_2016_1769_MOESM2_ESM.doc]

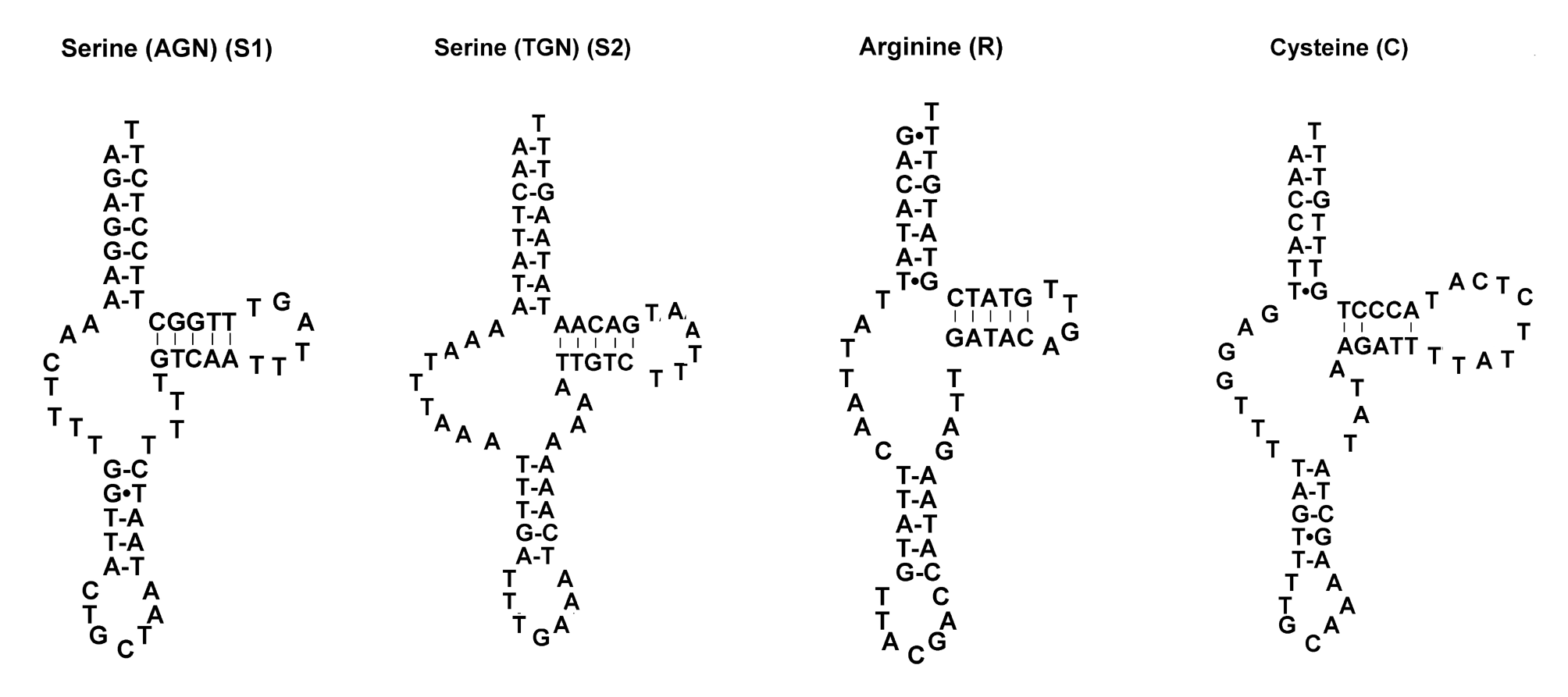


**Additional file 2: Figure S1.** Deduced secondary structures for the four tRNA genes of *Cladotaenia* *vulturi* mt genome. Only these four tRNA genes lack typical cloverleaf structure
