# Supplementary material for: The complete mitochondrial genome of the tapeworm Cladotaenia vulturi (Cestoda: Paruterinidae): gene arrangement and phylogenetic relationships with other cestodes
Source: Parasit Vectors. 2016 Aug 31;9(1):475. doi: 10.1186/s13071-016-1769-x (PMC5006517; doi:10.1186/s13071-016-1769-x)
Supplement: Additional file 3: — Figure S2. Putative secondary structures of the two non-coding regions in Cladotaenia vulturi mt genome. a The long non-coding region (NC1) located between trnY and trnS2. b The short non-coding region (NC2) between nad5 and trnG. (DOC 102 kb) [file 13071_2016_1769_MOESM3_ESM.doc]

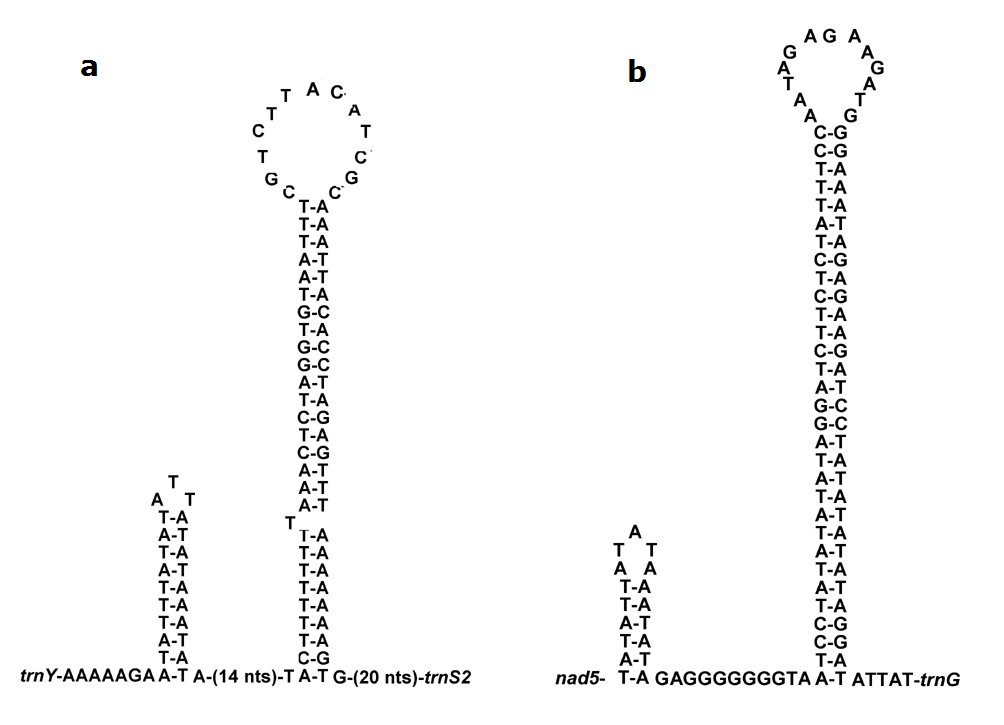


**Additional file 3: Figure S2.** Putative secondary structures of the two non-coding regions in *Cladotaenia* *vulturi* mt genome. **a** The long non-coding region (NC1) located between *trnY* and *trnS2*. **b** The short non-coding region (NC2) between *nad5* and *trnG*
