# Supplementary material for: The complete mitochondrial genome of the tapeworm Cladotaenia vulturi (Cestoda: Paruterinidae): gene arrangement and phylogenetic relationships with other cestodes
Source: Parasit Vectors. 2016 Aug 31;9(1):475. doi: 10.1186/s13071-016-1769-x (PMC5006517; doi:10.1186/s13071-016-1769-x)
Supplement: Additional file 4: — Table S2. Codon frequencies (N) and relative synonymous codon usage (RSCU) values in 12 mt protein-coding genes of cestode species. (DOC 1068 kb) [file 13071_2016_1769_MOESM4_ESM.doc]

**Additional file 4: Table S2.** Nucleotide codon usage for 12 protein-coding genes of the mitochondrial genome of cestode species

| **Amino acid** | **Codon** | **N** | **RSCU** | **Amino acid** | **Codon** | **N** | **RSCU** | **Amino acid** | **Codon** | **N** | **RSCU** | **Amino acid** | **Codon** | **N** | **RSCU** |
| --- | --- | --- | --- | --- | --- | --- | --- | --- | --- | --- | --- | --- | --- | --- | --- |
| *Taenia solium* | | | | | | | | | | | | | | | |
| Phe | UUU | 390 | 1.91 | Ser | UCU | 98 | 2.02 | Tyr | UAU | 179 | 1.82 | Cys | UGU | 128 | 1.86 |
| Phe | UUC | 18 | 0.09 | Ser | UCC | 3 | 0.06 | Tyr | UAC | 18 | 0.18 | Cys | UGC | 10 | 0.14 |
| Leu | UUA | 261 | 3.18 | Ser | UCA | 48 | 0.99 | Ter | UAA | 5 | 0.22 | Ter | UGA | 57 | 2.51 |
| Leu | UUG | 172 | 2.10 | Ser | UCG | 16 | 0.33 | Ter | UAG | 6 | 0.26 | Trp | UGG | 33 | 1.00 |
| Leu | CUU | 30 | 0.37 | Pro | CCU | 36 | 2.03 | His | CAU | 51 | 1.82 | Arg | CGU | 37 | 1.75 |
| Leu | CUC | 4 | 0.05 | Pro | CCC | 2 | 0.11 | His | CAC | 5 | 0.18 | Arg | CGC | 0 | 0 |
| Leu | CUA | 17 | 0.21 | Pro | CCA | 26 | 1.46 | Gln | CAA | 15 | 1.30 | Arg | CGA | 7 | 0.33 |
| Leu | CUG | 8 | 0.10 | Pro | CCG | 7 | 0.39 | Gln | CAG | 8 | 0.70 | Arg | CGG | 5 | 0.24 |
| Ile | AUU | 165 | 1.59 | Thr | ACU | 61 | 2.57 | Asn | AAU | 67 | 1.89 | Ser | AGU | 120 | 2.47 |
| Ile | AUC | 16 | 0.15 | Thr | ACC | 2 | 0.08 | Asn | AAC | 4 | 0.11 | Ser | AGC | 6 | 0.12 |
| Ile | AUA | 131 | 1.26 | Thr | ACA | 22 | 0.93 | Lys | AAA | 54 | 1.06 | Arg | AGA | 52 | 2.46 |
| Met | AUG | 97 | 1.00 | Thr | ACG | 10 | 0.42 | Lys | AAG | 48 | 0.94 | Arg | AGG | 26 | 1.23 |
| Val | GUU | 178 | 2.08 | Ala | GCU | 52 | 2.57 | Asp | GAU | 81 | 1.93 | Gly | GGU | 104 | 2.16 |
| Val | GUC | 16 | 0.19 | Ala | GCC | 3 | 0.15 | Asp | GAC | 3 | 0.07 | Gly | GGC | 11 | 0.23 |
| Val | GUA | 88 | 1.03 | Ala | GCA | 16 | 0.79 | Glu | GAA | 38 | 1.12 | Gly | GGA | 41 | 0.85 |
| Val | GUG | 60 | 0.70 | Ala | GCG | 10 | 0.49 | Glu | GAG | 30 | 0.88 | Gly | GGG | 37 | 0.77 |
| *Taenia saginata* | | | | | | | | | | | | | | | |
| Phe | UUU | 398 | 1.93 | Ser | UCU | 104 | 2.22 | Tyr | UAU | 191 | 1.93 | Cys | UGU | 131 | 1.85 |
| Phe | UUC | 15 | 0.07 | Ser | UCC | 0 | 0.00 | Tyr | UAC | 7 | 0.07 | Cys | UGC | 11 | 0.15 |
| Leu | UUA | 256 | 3.12 | Ser | UCA | 44 | 0.94 | Ter | UAA | 0 | 0.00 | Ter | UGA | 44 | 3.00 |
| Leu | UUG | 189 | 2.30 | Ser | UCG | 20 | 0.43 | Ter | UAG | 0 | 0.00 | Trp | UGG | 46 | 1.00 |
| Leu | CUU | 27 | 0.33 | Pro | CCU | 50 | 2.82 | His | CAU | 54 | 1.96 | Arg | CGU | 39 | 1.79 |
| Leu | CUC | 3 | 0.04 | Pro | CCC | 4 | 0.23 | His | CAC | 1 | 0.04 | Arg | CGC | 2 | 0.09 |
| Leu | CUA | 10 | 0.12 | Pro | CCA | 12 | 0.68 | Gln | CAA | 14 | 1.17 | Arg | CGA | 6 | 0.27 |
| Leu | CUG | 7 | 0.09 | Pro | CCG | 5 | 0.28 | Gln | CAG | 10 | 0.83 | Arg | CGG | 4 | 0.18 |
| Ile | AUU | 170 | 1.71 | Thr | ACU | 68 | 2.69 | Asn | AAU | 63 | 1.80 | Ser | AGU | 111 | 2.37 |
| Ile | AUC | 9 | 0.09 | Thr | ACC | 4 | 0.16 | Asn | AAC | 7 | 0.20 | Ser | AGC | 2 | 0.04 |
| Ile | AUA | 120 | 1.20 | Thr | ACA | 17 | 0.67 | Lys | AAA | 56 | 1.07 | Arg | AGA | 54 | 2.47 |
| Met | AUG | 88 | 1.00 | Thr | ACG | 12 | 0.48 | Lys | AAG | 49 | 0.93 | Arg | AGG | 26 | 1.19 |
| Val | GUU | 189 | 2.21 | Ala | GCU | 61 | 2.84 | Asp | GAU | 75 | 1.95 | Gly | GGU | 141 | 2.67 |
| Val | GUC | 6 | 0.07 | Ala | GCC | 2 | 0.09 | Asp | GAC | 2 | 0.05 | Gly | GGC | 2 | 0.04 |
| Val | GUA | 67 | 0.78 | Ala | GCA | 14 | 0.65 | Glu | GAA | 30 | 0.88 | Gly | GGA | 33 | 0.63 |
| Val | GUG | 80 | 0.94 | Ala | GCG | 9 | 0.42 | Glu | GAG | 38 | 1.12 | Gly | GGG | 35 | 0.66 |
| *Taenia asiatica* | | | | | | | | | | | | | | | |
| Phe | UUU | 391 | 1.90 | Ser | UCU | 98 | 2.12 | Tyr | UAU | 191 | 1.90 | Cys | UGU | 130 | 1.86 |
| Phe | UUC | 20 | 0.10 | Ser | UCC | 4 | 0.09 | Tyr | UAC | 10 | 0.10 | Cys | UGC | 10 | 0.14 |
| Leu | UUA | 243 | 2.98 | Ser | UCA | 45 | 0.97 | Ter | UAA | 6 | 0.33 | Ter | UGA | 44 | 2.40 |
| Leu | UUG | 196 | 2.40 | Ser | UCG | 18 | 0.39 | Ter | UAG | 5 | 0.27 | Trp | UGG | 47 | 1.00 |
| Leu | CUU | 31 | 0.38 | Pro | CCU | 46 | 2.63 | His | CAU | 51 | 1.96 | Arg | CGU | 40 | 1.82 |
| Leu | CUC | 0 | 0.00 | Pro | CCC | 5 | 0.29 | His | CAC | 1 | 0.04 | Arg | CGC | 1 | 0.05 |
| Leu | CUA | 10 | 0.12 | Pro | CCA | 9 | 0.51 | Gln | CAA | 12 | 1.04 | Arg | CGA | 7 | 0.32 |
| Leu | CUG | 10 | 0.12 | Pro | CCG | 10 | 0.57 | Gln | CAG | 11 | 0.96 | Arg | CGG | 4 | 0.18 |
| Ile | AUU | 167 | 1.68 | Thr | ACU | 69 | 2.79 | Asn | AAU | 64 | 1.80 | Ser | AGU | 110 | 2.37 |
| Ile | AUC | 10 | 0.10 | Thr | ACC | 4 | 0.61 | Asn | AAC | 7 | 0.20 | Ser | AGC | 3 | 0.06 |
| Ile | AUA | 121 | 1.22 | Thr | ACA | 15 | 0.61 | Lys | AAA | 55 | 1.05 | Arg | AGA | 58 | 2.64 |
| Met | AUG | 97 | 1.00 | Thr | ACG | 11 | 0.44 | Lys | AAG | 50 | 0.95 | Arg | AGG | 22 | 1.00 |
| Val | GUU | 193 | 2.20 | Ala | GCU | 60 | 2.82 | Asp | GAU | 75 | 1.95 | Gly | GGU | 137 | 2.60 |
| Val | GUC | 9 | 0.10 | Ala | GCC | 1 | 0.05 | Asp | GAC | 2 | 0.05 | Gly | GGC | 4 | 0.08 |
| Val | GUA | 81 | 0.92 | Ala | GCA | 12 | 0.56 | Glu | GAA | 29 | 0.85 | Gly | GGA | 33 | 0.63 |
| Val | GUG | 68 | 0.77 | Ala | GCG | 12 | 0.56 | Glu | GAG | 39 | 1.15 | Gly | GGG | 37 | 0.70 |
| *Taenia ovis* | | | | | | | | | | | | | | | |
| Phe | UUU | 387 | 1.88 | Ser | UCU | 96 | 2.11 | Tyr | UAU | 181 | 1.85 | Cys | UGU | 131 | 1.87 |
| Phe | UUC | 25 | 0.12 | Ser | UCC | 6 | 0.13 | Tyr | UAC | 15 | 0.15 | Cys | UGC | 9 | 0.13 |
| Leu | UUA | 221 | 2.66 | Ser | UCA | 29 | 0.64 | Ter | UAA | 5 | 0.28 | Ter | UGA | 42 | 2.38 |
| Leu | UUG | 218 | 2.62 | Ser | UCG | 23 | 0.51 | Ter | UAG | 6 | 0.34 | Trp | UGG | 51 | 1.00 |
| Leu | CUU | 36 | 0.43 | Pro | CCU | 47 | 2.58 | His | CAU | 50 | 1.85 | Arg | CGU | 41 | 1.84 |
| Leu | CUC | 2 | 0.02 | Pro | CCC | 3 | 0.16 | His | CAC | 4 | 0.15 | Arg | CGC | 2 | 0.09 |
| Leu | CUA | 4 | 0.05 | Pro | CCA | 13 | 0.71 | Gln | CAA | 13 | 1.13 | Arg | CGA | 4 | 0.18 |
| Leu | CUG | 18 | 0.22 | Pro | CCG | 10 | 0.55 | Gln | CAG | 10 | 0.87 | Arg | CGG | 7 | 0.31 |
| Ile | AUU | 156 | 1.65 | Thr | ACU | 66 | 2.61 | Asn | AAU | 64 | 1.88 | Ser | AGU | 110 | 2.42 |
| Ile | AUC | 13 | 0.14 | Thr | ACC | 5 | 0.20 | Asn | AAC | 4 | 0.12 | Ser | AGC | 9 | 0.20 |
| Ile | AUA | 114 | 1.21 | Thr | ACA | 15 | 0.59 | Lys | AAA | 54 | 1.07 | Arg | AGA | 31 | 1.39 |
| Met | AUG | 96 | 1.00 | Thr | ACG | 15 | 0.59 | Lys | AAG | 47 | 0.93 | Arg | AGG | 49 | 2.19 |
| Val | GUU | 183 | 2.05 | Ala | GCU | 58 | 2.58 | Asp | GAU | 70 | 1.82 | Gly | GGU | 147 | 2.75 |
| Val | GUC | 12 | 0.13 | Ala | GCC | 8 | 0.36 | Asp | GAC | 7 | 0.18 | Gly | GGC | 7 | 0.13 |
| Val | GUA | 66 | 0.74 | Ala | GCA | 13 | 0.58 | Glu | GAA | 27 | 0.81 | Gly | GGA | 25 | 0.47 |
| Val | GUG | 96 | 1.08 | Ala | GCG | 11 | 0.49 | Glu | GAG | 40 | 1.19 | Gly | GGG | 35 | 0.65 |
| *Echinococcus multilocularis* | | | | | | | | | | | | | | | |
| Phe | UUU | 402 | 1.94 | Ser | UCU | 99 | 2.13 | Tyr | UAU | 193 | 1.82 | Cys | UGU | 144 | 1.95 |
| Phe | UUC | 13 | 0.06 | Ser | UCC | 2 | 0.04 | Tyr | UAC | 19 | 0.18 | Cys | UGC | 4 | 0.05 |
| Leu | UUA | 181 | 2.19 | Ser | UCA | 36 | 0.77 | Ter | UAA | 4 | 0.27 | Ter | UGA | 33 | 2.20 |
| Leu | UUG | 271 | 3.27 | Ser | UCG | 21 | 0.45 | Ter | UAG | 8 | 0.53 | Trp | UGG | 59 | 1.00 |
| Leu | CUU | 24 | 0.29 | Pro | CCU | 45 | 2.54 | His | CAU | 46 | 1.88 | Arg | CGU | 34 | 1.76 |
| Leu | CUC | 0 | 0.00 | Pro | CCC | 0 | 0.00 | His | CAC | 3 | 0.12 | Arg | CGC | 1 | 0.05 |
| Leu | CUA | 7 | 0.08 | Pro | CCA | 13 | 0.73 | Gln | CAA | 10 | 0.83 | Arg | CGA | 7 | 0.36 |
| Leu | CUG | 14 | 0.17 | Pro | CCG | 13 | 0.73 | Gln | CAG | 14 | 1.17 | Arg | CGG | 9 | 0.47 |
| Ile | AUU | 148 | 2.03 | Thr | ACU | 65 | 2.95 | Asn | AAU | 87 | 1.98 | Ser | AGU | 114 | 2.45 |
| Ile | AUC | 4 | 0.05 | Thr | ACC | 0 | 0.00 | Asn | AAC | 1 | 0.02 | Ser | AGC | 7 | 0.15 |
| Ile | AUA | 67 | 0.92 | Thr | ACA | 9 | 0.41 | Lys | AAA | 18 | 0.59 | Arg | AGA | 32 | 1.66 |
| Met | AUG | 90 | 1.00 | Thr | ACG | 14 | 0.64 | Lys | AAG | 43 | 1.41 | Arg | AGG | 33 | 1.71 |
| Val | GUU | 238 | 2.18 | Ala | GCU | 59 | 2.88 | Asp | GAU | 74 | 1.95 | Gly | GGU | 155 | 2.66 |
| Val | GUC | 9 | 0.08 | Ala | GCC | 4 | 0.20 | Asp | GAC | 2 | 0.05 | Gly | GGC | 5 | 0.09 |
| Val | GUA | 75 | 0.69 | Ala | GCA | 8 | 0.39 | Glu | GAA | 17 | 0.54 | Gly | GGA | 19 | 0.33 |
| Val | GUG | 114 | 1.05 | Ala | GCG | 11 | 0.54 | Glu | GAG | 46 | 1.46 | Gly | GGG | 54 | 0.93 |
| *Echinococcus granulosus* | | | | | | | | | | | | | | | |
| Phe | UUU | 378 | 1.90 | Ser | UCU | 98 | 2.24 | Tyr | UAU | 206 | 1.90 | Cys | UGU | 140 | 1.88 |
| Phe | UUC | 20 | 0.10 | Ser | UCC | 6 | 0.14 | Tyr | UAC | 11 | 0.10 | Cys | UGC | 9 | 0.12 |
| Leu | UUA | 146 | 1.72 | Ser | UCA | 20 | 0.46 | Ter | UAA | 4 | 0.29 | Ter | UGA | 30 | 2.14 |
| Leu | UUG | 305 | 3.60 | Ser | UCG | 37 | 0.84 | Ter | UAG | 8 | 0.57 | Trp | UGG | 67 | 1.00 |
| Leu | CUU | 32 | 0.38 | Pro | CCU | 36 | 2.09 | His | CAU | 46 | 1.84 | Arg | CGU | 35 | 1.67 |
| Leu | CUC | 2 | 0.02 | Pro | CCC | 2 | 0.12 | His | CAC | 4 | 0.16 | Arg | CGC | 2 | 0.10 |
| Leu | CUA | 8 | 0.09 | Pro | CCA | 14 | 0.81 | Gln | CAA | 8 | 0.64 | Arg | CGA | 1 | 0.05 |
| Leu | CUG | 15 | 0.18 | Pro | CCG | 17 | 0.99 | Gln | CAG | 17 | 1.36 | Arg | CGG | 12 | 0.57 |
| Ile | AUU | 141 | 2.12 | Thr | ACU | 60 | 2.70 | Asn | AAU | 77 | 1.90 | Ser | AGU | 93 | 2.12 |
| Ile | AUC | 9 | 0.14 | Thr | ACC | 2 | 0.09 | Asn | AAC | 4 | 0.10 | Ser | AGC | 9 | 0.21 |
| Ile | AUA | 50 | 0.75 | Thr | ACA | 8 | 0.36 | Lys | AAA | 16 | 0.55 | Arg | AGA | 27 | 1.29 |
| Met | AUG | 92 | 1.00 | Thr | ACG | 19 | 0.85 | Lys | AAG | 42 | 1.45 | Arg | AGG | 49 | 2.33 |
| Val | GUU | 268 | 2.29 | Ala | GCU | 48 | 2.40 | Asp | GAU | 80 | 1.95 | Gly | GGU | 147 | 2.42 |
| Val | GUC | 12 | 0.10 | Ala | GCC | 9 | 0.45 | Asp | GAC | 2 | 0.05 | Gly | GGC | 12 | 0.20 |
| Val | GUA | 48 | 0.41 | Ala | GCA | 7 | 0.35 | Glu | GAA | 17 | 0.52 | Gly | GGA | 22 | 0.36 |
| Val | GUG | 141 | 1.20 | Ala | GCG | 16 | 0.80 | Glu | GAG | 48 | 1.48 | Gly | GGG | 62 | 1.02 |
| *Cladotaenia vulturi* | | | | | | | | | | | | | | | |
| Phe | UUU | 363 | 1.89 | Ser | UCU | 121 | 2.52 | Tyr | UAU | 206 | 1.94 | Cys | UGU | 137 | 1.90 |
| Phe | UUC | 22 | 0.11 | Ser | UCC | 1 | 0.02 | Tyr | UAC | 6 | 0.06 | Cys | UGC | 7 | 0.10 |
| Leu | UUA | 311 | 3.78 | Ser | UCA | 43 | 0.90 | Ter | UAA | 8 | 0.37 | Ter | UGA | 54 | 2.49 |
| Leu | UUG | 130 | 1.58 | Ser | UCG | 10 | 0.21 | Ter | UAG | 3 | 0.14 | Trp | UGG | 29 | 1.00 |
| Leu | CUU | 36 | 0.44 | Pro | CCU | 35 | 1.89 | His | CAU | 57 | 1.90 | Arg | CGU | 31 | 1.54 |
| Leu | CUC | 0 | 0.00 | Pro | CCC | 1 | 0.05 | His | CAC | 3 | 0.10 | Arg | CGC | 3 | 0.15 |
| Leu | CUA | 17 | 0.21 | Pro | CCA | 27 | 1.46 | Gln | CAA | 13 | 1.08 | Arg | CGA | 7 | 0.35 |
| Leu | CUG | 0 | 0.00 | Pro | CCG | 11 | 0.59 | Gln | CAG | 11 | 0.92 | Arg | CGG | 5 | 0.25 |
| Ile | AUU | 201 | 1.53 | Thr | ACU | 64 | 2.59 | Asn | AAU | 86 | 1.87 | Ser | AGU | 107 | 2.23 |
| Ile | AUC | 10 | 0.08 | Thr | ACC | 3 | 0.12 | Asn | AAC | 6 | 0.13 | Ser | AGC | 6 | 0.13 |
| Ile | AUA | 182 | 1.39 | Thr | ACA | 28 | 1.13 | Lys | AAA | 68 | 1.25 | Arg | AGA | 61 | 3.02 |
| Met | AUG | 92 | 1.00 | Thr | ACG | 4 | 0.16 | Lys | AAG | 41 | 0.75 | Arg | AGG | 14 | 0.69 |
| Val | GUU | 142 | 2.09 | Ala | GCU | 45 | 2.47 | Asp | GAU | 79 | 1.95 | Gly | GGU | 100 | 2.14 |
| Val | GUC | 3 | 0.04 | Ala | GCC | 4 | 0.22 | Asp | GAC | 2 | 0.05 | Gly | GGC | 2 | 0.04 |
| Val | GUA | 89 | 1.31 | Ala | GCA | 18 | 0.99 | Glu | GAA | 38 | 1.25 | Gly | GGA | 49 | 1.05 |
| Val | GUG | 38 | 0.56 | Ala | GCG | 6 | 0.33 | Glu | GAG | 23 | 0.75 | Gly | GGG | 36 | 0.77 |
| *Versteria mustelae* | | | | | | | | | | | | | | | |
| Phe | UUU | 376 | 1.88 | Ser | UCU | 145 | 2.90 | Tyr | UAU | 202 | 1.84 | Cys | UGU | 131 | 1.86 |
| Phe | UUC | 23 | 0.12 | Ser | UCC | 7 | 0.14 | Tyr | UAC | 17 | 0.16 | Cys | UGC | 10 | 0.14 |
| Leu | UUA | 209 | 2.48 | Ser | UCA | 23 | 0.46 | Ter | UAA | 0 | 0.00 | Ter | UGA | 39 | 3.00 |
| Leu | UUG | 249 | 2.96 | Ser | UCG | 12 | 0.24 | Ter | UAG | 0 | 0.00 | Trp | UGG | 48 | 1.00 |
| Leu | CUU | 23 | 0.27 | Pro | CCU | 51 | 3.00 | His | CAU | 50 | 2.00 | Arg | CGU | 32 | 1.63 |
| Leu | CUC | 0 | 0.00 | Pro | CCC | 2 | 0.12 | His | CAC | 0 | 0.00 | Arg | CGC | 0 | 0.00 |
| Leu | CUA | 18 | 0.21 | Pro | CCA | 8 | 0.47 | Gln | CAA | 9 | 0.78 | Arg | CGA | 4 | 0.20 |
| Leu | CUG | 6 | 0.07 | Pro | CCG | 7 | 0.41 | Gln | CAG | 14 | 1.22 | Arg | CGG | 12 | 0.61 |
| Ile | AUU | 125 | 1.29 | Thr | ACU | 82 | 3.53 | Asn | AAU | 82 | 1.91 | Ser | AGU | 110 | 2.20 |
| Ile | AUC | 7 | 0.07 | Thr | ACC | 1 | 0.04 | Asn | AAC | 4 | 0.09 | Ser | AGC | 3 | 0.06 |
| Ile | AUA | 158 | 1.63 | Thr | ACA | 5 | 0.22 | Lys | AAA | 42 | 0.97 | Arg | AGA | 39 | 1.98 |
| Met | AUG | 92 | 1.00 | Thr | ACG | 5 | 0.22 | Lys | AAG | 45 | 1.03 | Arg | AGG | 31 | 1.58 |
| Val | GUU | 209 | 2.36 | Ala | GCU | 48 | 2.87 | Asp | GAU | 76 | 1.90 | Gly | GGU | 161 | 2.89 |
| Val | GUC | 8 | 0.09 | Ala | GCC | 4 | 0.24 | Asp | GAC | 4 | 0.10 | Gly | GGC | 3 | 0.05 |
| Val | GUA | 61 | 0.69 | Ala | GCA | 8 | 0.48 | Glu | GAA | 27 | 0.90 | Gly | GGA | 19 | 0.34 |
| Val | GUG | 76 | 0.86 | Ala | GCG | 7 | 0.42 | Glu | GAG | 33 | 1.10 | Gly | GGG | 40 | 0.72 |
| *Pseudanoplocephala crawfordi* | | | | | | | | | | | | | | | |
| Phe | UUU | 388 | 1.88 | Ser | UCU | 115 | 2.54 | Tyr | UAU | 189 | 1.77 | Cys | UGU | 121 | 1.83 |
| Phe | UUC | 25 | 0.12 | Ser | UCC | 4 | 0.09 | Tyr | UAC | 24 | 0.23 | Cys | UGC | 11 | 0.17 |
| Leu | UUA | 235 | 2.69 | Ser | UCA | 31 | 0.68 | Ter | UAA | 0 | 0.00 | Ter | UGA | 56 | 3.00 |
| Leu | UUG | 195 | 2.23 | Ser | UCG | 13 | 0.29 | Ter | UAG | 0 | 0.00 | Trp | UGG | 39 | 1.00 |
| Leu | CUU | 42 | 0.48 | Pro | CCU | 33 | 1.65 | His | CAU | 43 | 1.59 | Arg | CGU | 40 | 1.94 |
| Leu | CUC | 5 | 0.06 | Pro | CCC | 3 | 0.15 | His | CAC | 11 | 0.41 | Arg | CGC | 1 | 0.05 |
| Leu | CUA | 32 | 0.37 | Pro | CCA | 19 | 0.95 | Gln | CAA | 9 | 0.82 | Arg | CGA | 5 | 0.24 |
| Leu | CUG | 15 | 0.17 | Pro | CCG | 25 | 1.25 | Gln | CAG | 13 | 1.18 | Arg | CGG | 2 | 0.10 |
| Ile | AUU | 173 | 1.81 | Thr | ACU | 65 | 2.39 | Asn | AAU | 70 | 1.89 | Ser | AGU | 92 | 2.03 |
| Ile | AUC | 9 | 0.09 | Thr | ACC | 3 | 0.11 | Asn | AAC | 4 | 0.11 | Ser | AGC | 17 | 0.38 |
| Ile | AUA | 104 | 1.09 | Thr | ACA | 32 | 1.17 | Lys | AAA | 49 | 0.97 | Arg | AGA | 47 | 2.27 |
| Met | AUG | 82 | 1.00 | Thr | ACG | 9 | 0.33 | Lys | AAG | 52 | 1.03 | Arg | AGG | 29 | 1.40 |
| Val | GUU | 148 | 2.00 | Ala | GCU | 46 | 1.80 | Asp | GAU | 69 | 1.86 | Gly | GGU | 96 | 1.79 |
| Val | GUC | 9 | 0.12 | Ala | GCC | 7 | 0.27 | Asp | GAC | 5 | 0.14 | Gly | GGC | 19 | 0.35 |
| Val | GUA | 57 | 0.77 | Ala | GCA | 24 | 0.94 | Glu | GAA | 40 | 1.13 | Gly | GGA | 41 | 0.76 |
| Val | GUG | 82 | 1.11 | Ala | GCG | 25 | 0.98 | Glu | GAG | 31 | 0.87 | Gly | GGG | 59 | 1.10 |
| *Drepanidotaenia lanceolata* | | | | | | | | | | | | | | | |
| Phe | UUU | 392 | 1.86 | Ser | UCU | 121 | 2.66 | Tyr | UAU | 193 | 1.80 | Cys | UGU | 108 | 1.79 |
| Phe | UUC | 29 | 0.14 | Ser | UCC | 7 | 0.15 | Tyr | UAC | 22 | 0.20 | Cys | UGC | 13 | 0.21 |
| Leu | UUA | 206 | 2.50 | Ser | UCA | 40 | 0.88 | Ter | UAA | 0 | 0.00 | Ter | UGA | 41 | 3.00 |
| Leu | UUG | 171 | 2.07 | Ser | UCG | 15 | 0.33 | Ter | UAG | 0 | 0.00 | Trp | UGG | 41 | 1.00 |
| Leu | CUU | 67 | 0.81 | Pro | CCU | 58 | 2.83 | His | CAU | 46 | 1.88 | Arg | CGU | 39 | 1.70 |
| Leu | CUC | 3 | 0.04 | Pro | CCC | 2 | 0.10 | His | CAC | 3 | 0.12 | Arg | CGC | 1 | 0.04 |
| Leu | CUA | 24 | 0.29 | Pro | CCA | 8 | 0.39 | Gln | CAA | 8 | 0.59 | Arg | CGA | 6 | 0.26 |
| Leu | CUG | 24 | 0.29 | Pro | CCG | 14 | 0.68 | Gln | CAG | 19 | 1.41 | Arg | CGG | 8 | 0.35 |
| Ile | AUU | 154 | 1.53 | Thr | ACU | 79 | 2.68 | Asn | AAU | 63 | 1.77 | Ser | AGU | 80 | 1.76 |
| Ile | AUC | 6 | 0.06 | Thr | ACC | 2 | 0.07 | Asn | AAC | 8 | 0.23 | Ser | AGC | 10 | 0.22 |
| Ile | AUA | 142 | 1.41 | Thr | ACA | 25 | 0.85 | Lys | AAA | 64 | 1.03 | Arg | AGA | 49 | 2.13 |
| Met | AUG | 83 | 1.00 | Thr | ACG | 12 | 0.41 | Lys | AAG | 60 | 0.97 | Arg | AGG | 35 | 1.52 |
| Val | GUU | 147 | 2.00 | Ala | GCU | 62 | 2.51 | Asp | GAU | 58 | 1.73 | Gly | GGU | 110 | 2.22 |
| Val | GUC | 11 | 0.15 | Ala | GCC | 10 | 0.40 | Asp | GAC | 9 | 0.27 | Gly | GGC | 12 | 0.24 |
| Val | GUA | 65 | 0.88 | Ala | GCA | 9 | 0.36 | Glu | GAA | 27 | 0.75 | Gly | GGA | 20 | 0.40 |
| Val | GUG | 71 | 0.97 | Ala | GCG | 18 | 0.73 | Glu | GAG | 45 | 1.25 | Gly | GGG | 56 | 1.13 |
| *Hymenolepis nana* | | | | | | | | | | | | | | | |
| Phe | UUU | 369 | 1.88 | Ser | UCU | 124 | 2.43 | Tyr | UAU | 176 | 1.74 | Cys | UGU | 115 | 1.83 |
| Phe | UUC | 23 | 0.12 | Ser | UCC | 4 | 0.08 | Tyr | UAC | 26 | 0.26 | Cys | UGC | 11 | 0.17 |
| Leu | UUA | 291 | 3.52 | Ser | UCA | 46 | 0.90 | Ter | UAA | 0 | 0.00 | Ter | UGA | 41 | 3.00 |
| Leu | UUG | 142 | 1.72 | Ser | UCG | 17 | 0.33 | Ter | UAG | 0 | 0.00 | Trp | UGG | 44 | 1.00 |
| Leu | CUU | 33 | 0.40 | Pro | CCU | 43 | 2.15 | His | CAU | 49 | 1.85 | Arg | CGU | 31 | 1.45 |
| Leu | CUC | 0 | 0.00 | Pro | CCC | 3 | 0.15 | His | CAC | 4 | 0.15 | Arg | CGC | 0 | 0.00 |
| Leu | CUA | 22 | 0.27 | Pro | CCA | 26 | 1.30 | Gln | CAA | 9 | 0.78 | Arg | CGA | 13 | 0.61 |
| Leu | CUG | 8 | 0.10 | Pro | CCG | 8 | 0.40 | Gln | CAG | 14 | 1.22 | Arg | CGG | 6 | 0.28 |
| Ile | AUU | 166 | 1.37 | Thr | ACU | 75 | 2.78 | Asn | AAU | 87 | 1.85 | Ser | AGU | 102 | 2.00 |
| Ile | AUC | 13 | 0.11 | Thr | ACC | 1 | 0.04 | Asn | AAC | 7 | 0.15 | Ser | AGC | 13 | 0.25 |
| Ile | AUA | 184 | 1.52 | Thr | ACA | 23 | 0.85 | Lys | AAA | 54 | 1.04 | Arg | AGA | 54 | 2.53 |
| Met | AUG | 83 | 1.00 | Thr | ACG | 9 | 0.33 | Lys | AAG | 50 | 0.96 | Arg | AGG | 24 | 1.13 |
| Val | GUU | 139 | 2.00 | Ala | GCU | 49 | 2.36 | Asp | GAU | 66 | 1.78 | Gly | GGU | 114 | 2.51 |
| Val | GUC | 7 | 0.10 | Ala | GCC | 5 | 0.24 | Asp | GAC | 8 | 0.22 | Gly | GGC | 10 | 0.22 |
| Val | GUA | 90 | 1.29 | Ala | GCA | 22 | 1.06 | Glu | GAA | 38 | 1.17 | Gly | GGA | 29 | 0.64 |
| Val | GUG | 42 | 0.60 | Ala | GCG | 7 | 0.34 | Glu | GAG | 27 | 0.83 | Gly | GGG | 29 | 0.64 |
| *Hymenolepis diminuta* | | | | | | | | | | | | | | | |
| Phe | UUU | 392 | 1.88 | Ser | UCU | 112 | 2.48 | Tyr | UAU | 181 | 1.68 | Cys | UGU | 107 | 1.63 |
| Phe | UUC | 24 | 0.12 | Ser | UCC | 7 | 0.15 | Tyr | UAC | 34 | 0.32 | Cys | UGC | 24 | 0.37 |
| Leu | UUA | 300 | 3.56 | Ser | UCA | 32 | 0.71 | Ter | UAA | 0 | 0.00 | Ter | UGA | 57 | 3.00 |
| Leu | UUG | 119 | 1.41 | Ser | UCG | 19 | 0.42 | Ter | UAG | 0 | 0.00 | Trp | UGG | 31 | 1.00 |
| Leu | CUU | 42 | 0.50 | Pro | CCU | 52 | 2.60 | His | CAU | 42 | 1.58 | Arg | CGU | 40 | 1.66 |
| Leu | CUC | 4 | 0.05 | Pro | CCC | 6 | 0.30 | His | CAC | 11 | 0.42 | Arg | CGC | 1 | 0.04 |
| Leu | CUA | 27 | 0.32 | Pro | CCA | 19 | 0.95 | Gln | CAA | 7 | 0.64 | Arg | CGA | 10 | 0.41 |
| Leu | CUG | 14 | 0.17 | Pro | CCG | 3 | 0.15 | Gln | CAG | 15 | 1.36 | Arg | CGG | 1 | 0.04 |
| Ile | AUU | 171 | 1.74 | Thr | ACU | 60 | 2.18 | Asn | AAU | 70 | 1.71 | Ser | AGU | 87 | 1.93 |
| Ile | AUC | 16 | 0.16 | Thr | ACC | 5 | 0.18 | Asn | AAC | 12 | 0.29 | Ser | AGC | 14 | 0.31 |
| Ile | AUA | 108 | 1.10 | Thr | ACA | 28 | 1.02 | Lys | AAA | 54 | 1.08 | Arg | AGA | 56 | 2.32 |
| Met | AUG | 83 | 1.00 | Thr | ACG | 17 | 0.62 | Lys | AAG | 46 | 0.92 | Arg | AGG | 37 | 1.53 |
| Val | GUU | 143 | 1.93 | Ala | GCU | 47 | 1.88 | Asp | GAU | 57 | 1.70 | Gly | GGU | 84 | 1.77 |
| Val | GUC | 9 | 0.12 | Ala | GCC | 7 | 0.28 | Asp | GAC | 10 | 0.30 | Gly | GGC | 13 | 0.27 |
| Val | GUA | 85 | 1.14 | Ala | GCA | 30 | 1.20 | Glu | GAA | 34 | 0.93 | Gly | GGA | 43 | 0.91 |
| Val | GUG | 60 | 0.81 | Ala | GCG | 16 | 0.64 | Glu | GAG | 39 | 1.07 | Gly | GGG | 50 | 1.05 |
| *Anoplocephala magna* | | | | | | | | | | | | | | | |
| Phe | UUU | 417 | 1.92 | Ser | UCU | 102 | 2.40 | Tyr | UAU | 179 | 1.80 | Cys | UGU | 121 | 1.77 |
| Phe | UUC | 18 | 0.08 | Ser | UCC | 1 | 0.02 | Tyr | UAC | 20 | 0.20 | Cys | UGC | 16 | 0.23 |
| Leu | UUA | 260 | 3.01 | Ser | UCA | 35 | 0.82 | Ter | UAA | 0 | 0.00 | Ter | UGA | 61 | 3.00 |
| Leu | UUG | 186 | 2.15 | Ser | UCG | 21 | 0.49 | Ter | UAG | 0 | 0.00 | Trp | UGG | 30 | 1.00 |
| Leu | CUU | 43 | 0.50 | Pro | CCU | 44 | 2.35 | His | CAU | 45 | 1.80 | Arg | CGU | 31 | 1.31 |
| Leu | CUC | 1 | 0.01 | Pro | CCC | 2 | 0.11 | His | CAC | 5 | 0.20 | Arg | CGC | 0 | 0.00 |
| Leu | CUA | 14 | 0.16 | Pro | CCA | 22 | 1.17 | Gln | CAA | 9 | 0.72 | Arg | CGA | 10 | 0.42 |
| Leu | CUG | 15 | 0.17 | Pro | CCG | 7 | 0.37 | Gln | CAG | 16 | 1.28 | Arg | CGG | 6 | 0.25 |
| Ile | AUU | 167 | 1.81 | Thr | ACU | 64 | 2.81 | Asn | AAU | 67 | 1.91 | Ser | AGU | 89 | 2.09 |
| Ile | AUC | 10 | 0.11 | Thr | ACC | 3 | 0.13 | Asn | AAC | 3 | 0.09 | Ser | AGC | 7 | 0.16 |
| Ile | AUA | 100 | 1.08 | Thr | ACA | 17 | 0.75 | Lys | AAA | 51 | 0.95 | Arg | AGA | 53 | 2.24 |
| Met | AUG | 81 | 1.00 | Thr | ACG | 7 | 0.31 | Lys | AAG | 56 | 1.05 | Arg | AGG | 42 | 1.77 |
| Val | GUU | 188 | 2.22 | Ala | GCU | 55 | 2.68 | Asp | GAU | 66 | 1.83 | Gly | GGU | 104 | 1.87 |
| Val | GUC | 10 | 0.12 | Ala | GCC | 5 | 0.24 | Asp | GAC | 6 | 0.17 | Gly | GGC | 6 | 0.11 |
| Val | GUA | 79 | 0.93 | Ala | GCA | 14 | 0.68 | Glu | GAA | 33 | 0.93 | Gly | GGA | 58 | 1.05 |
| Val | GUG | 62 | 0.73 | Ala | GCG | 8 | 0.39 | Glu | GAG | 38 | 1.07 | Gly | GGG | 54 | 0.97 |
| *Anoplocephala perfoliata* | | | | | | | | | | | | | | | |
| Phe | UUU | 407 | 1.90 | Ser | UCU | 113 | 2.62 | Tyr | UAU | 169 | 1.77 | Cys | UGU | 121 | 1.79 |
| Phe | UUC | 21 | 0.10 | Ser | UCC | 0 | 0.00 | Tyr | UAC | 22 | 0.23 | Cys | UGC | 14 | 0.21 |
| Leu | UUA | 287 | 3.30 | Ser | UCA | 44 | 1.02 | Ter | UAA | 0 | 0.00 | Ter | UGA | 60 | 3.00 |
| Leu | UUG | 142 | 1.63 | Ser | UCG | 8 | 0.19 | Ter | UAG | 0 | 0.00 | Trp | UGG | 31 | 1.00 |
| Leu | CUU | 54 | 0.62 | Pro | CCU | 43 | 2.36 | His | CAU | 53 | 1.93 | Arg | CGU | 28 | 1.20 |
| Leu | CUC | 2 | 0.02 | Pro | CCC | 4 | 0.22 | His | CAC | 2 | 0.07 | Arg | CGC | 2 | 0.09 |
| Leu | CUA | 20 | 0.23 | Pro | CCA | 17 | 0.93 | Gln | CAA | 14 | 1.22 | Arg | CGA | 11 | 0.47 |
| Leu | CUG | 17 | 0.20 | Pro | CCG | 9 | 0.49 | Gln | CAG | 9 | 0.78 | Arg | CGG | 8 | 0.34 |
| Ile | AUU | 164 | 1.72 | Thr | ACU | 62 | 2.67 | Asn | AAU | 62 | 1.82 | Ser | AGU | 88 | 2.04 |
| Ile | AUC | 14 | 0.15 | Thr | ACC | 4 | 0.17 | Asn | AAC | 6 | 0.18 | Ser | AGC | 6 | 0.14 |
| Ile | AUA | 108 | 1.13 | Thr | ACA | 20 | 0.86 | Lys | AAA | 49 | 0.94 | Arg | AGA | 61 | 2.61 |
| Met | AUG | 76 | 1.00 | Thr | ACG | 7 | 0.30 | Lys | AAG | 55 | 1.06 | Arg | AGG | 30 | 1.29 |
| Val | GUU | 176 | 2.19 | Ala | GCU | 61 | 2.57 | Asp | GAU | 69 | 1.92 | Gly | GGU | 115 | 2.01 |
| Val | GUC | 9 | 0.11 | Ala | GCC | 7 | 0.29 | Asp | GAC | 3 | 0.08 | Gly | GGC | 7 | 0.12 |
| Val | GUA | 72 | 0.89 | Ala | GCA | 17 | 0.72 | Glu | GAA | 32 | 0.81 | Gly | GGA | 49 | 0.86 |
| Val | GUG | 65 | 0.81 | Ala | GCG | 10 | 0.42 | Glu | GAG | 47 | 1.19 | Gly | GGG | 58 | 1.01 |
| *Hydatigera taeniaeformis* | | | | | | | | | | | | | | | |
| Phe | UUU | 383 | 1.91 | Ser | UCU | 99 | 2.37 | Tyr | UAU | 174 | 1.78 | Cys | UGU | 116 | 1.81 |
| Phe | UUC | 17 | 0.09 | Ser | UCC | 3 | 0.07 | Tyr | UAC | 21 | 0.22 | Cys | UGC | 12 | 0.19 |
| Leu | UUA | 302 | 3.55 | Ser | UCA | 40 | 0.96 | Ter | UAA | 0 | 0.00 | Ter | UGA | 69 | 3.00 |
| Leu | UUG | 139 | 1.64 | Ser | UCG | 7 | 0.17 | Ter | UAG | 0 | 0.00 | Trp | UGG | 27 | 1.00 |
| Leu | CUU | 38 | 0.45 | Pro | CCU | 40 | 2.16 | His | CAU | 49 | 1.96 | Arg | CGU | 35 | 1.50 |
| Leu | CUC | 1 | 0.01 | Pro | CCC | 2 | 0.11 | His | CAC | 1 | 0.04 | Arg | CGC | 1 | 0.04 |
| Leu | CUA | 18 | 0.21 | Pro | CCA | 29 | 1.57 | Gln | CAA | 18 | 1.33 | Arg | CGA | 10 | 0.43 |
| Leu | CUG | 12 | 0.14 | Pro | CCG | 3 | 0.16 | Gln | CAG | 9 | 0.67 | Arg | CGG | 4 | 0.17 |
| Ile | AUU | 166 | 1.51 | Thr | ACU | 67 | 2.53 | Asn | AAU | 86 | 1.89 | Ser | AGU | 83 | 1.98 |
| Ile | AUC | 9 | 0.08 | Thr | ACC | 5 | 0.19 | Asn | AAC | 5 | 0.11 | Ser | AGC | 19 | 0.45 |
| Ile | AUA | 155 | 1.41 | Thr | ACA | 27 | 1.02 | Lys | AAA | 47 | 1.00 | Arg | AGA | 68 | 2.91 |
| Met | AUG | 87 | 1.00 | Thr | ACG | 7 | 0.26 | Lys | AAG | 47 | 1.00 | Arg | AGG | 22 | 0.94 |
| Val | GUU | 157 | 2.06 | Ala | GCU | 63 | 3.00 | Asp | GAU | 86 | 1.95 | Gly | GGU | 116 | 2.19 |
| Val | GUC | 8 | 0.10 | Ala | GCC | 4 | 0.19 | Asp | GAC | 2 | 0.05 | Gly | GGC | 9 | 0.17 |
| Val | GUA | 82 | 1.08 | Ala | GCA | 12 | 0.57 | Glu | GAA | 46 | 1.46 | Gly | GGA | 37 | 0.70 |
| Val | GUG | 58 | 0.76 | Ala | GCG | 5 | 0.24 | Glu | GAG | 17 | 0.54 | Gly | GGG | 50 | 0.94 |
| *Hydatigera parva* | | | | | | | | | | | | | | | |
| Phe | UUU | 370 | 1.90 | Ser | UCU | 103 | 2.24 | Tyr | UAU | 178 | 1.78 | Cys | UGU | 120 | 1.80 |
| Phe | UUC | 19 | 0.10 | Ser | UCC | 3 | 0.07 | Tyr | UAC | 22 | 0.22 | Cys | UGC | 13 | 0.20 |
| Leu | UUA | 285 | 3.37 | Ser | UCA | 44 | 0.96 | Ter | UAA | 0 | 0.00 | Ter | UGA | 60 | 3.00 |
| Leu | UUG | 165 | 1.95 | Ser | UCG | 19 | 0.41 | Ter | UAG | 0 | 0.00 | Trp | UGG | 37 | 1.00 |
| Leu | CUU | 31 | 0.37 | Pro | CCU | 37 | 2.06 | His | CAU | 47 | 1.92 | Arg | CGU | 25 | 1.11 |
| Leu | CUC | 2 | 0.02 | Pro | CCC | 3 | 0.17 | His | CAC | 2 | 0.08 | Arg | CGC | 1 | 0.04 |
| Leu | CUA | 11 | 0.13 | Pro | CCA | 25 | 1.39 | Gln | CAA | 11 | 0.88 | Arg | CGA | 10 | 0.44 |
| Leu | CUG | 13 | 0.15 | Pro | CCG | 7 | 0.39 | Gln | CAG | 14 | 1.12 | Arg | CGG | 10 | 0.44 |
| Ile | AUU | 170 | 1.66 | Thr | ACU | 61 | 2.54 | Asn | AAU | 75 | 1.83 | Ser | AGU | 95 | 2.07 |
| Ile | AUC | 12 | 0.12 | Thr | ACC | 3 | 0.13 | Asn | AAC | 7 | 0.17 | Ser | AGC | 12 | 0.26 |
| Ile | AUA | 125 | 1.22 | Thr | ACA | 19 | 0.79 | Lys | AAA | 53 | 1.05 | Arg | AGA | 60 | 2.67 |
| Met | AUG | 80 | 1.00 | Thr | ACG | 13 | 0.54 | Lys | AAG | 48 | 0.95 | Arg | AGG | 29 | 1.29 |
| Val | GUU | 182 | 2.07 | Ala | GCU | 52 | 2.67 | Asp | GAU | 79 | 1.95 | Gly | GGU | 121 | 2.33 |
| Val | GUC | 8 | 0.09 | Ala | GCC | 0 | 0.00 | Asp | GAC | 2 | 0.05 | Gly | GGC | 7 | 0.13 |
| Val | GUA | 80 | 0.91 | Ala | GCA | 16 | 0.82 | Glu | GAA | 36 | 1.01 | Gly | GGA | 26 | 0.50 |
| Val | GUG | 82 | 0.93 | Ala | GCG | 10 | 0.51 | Glu | GAG | 35 | 0.99 | Gly | GGG | 54 | 1.04 |
| *Hydatigera krepkogorski* | | | | | | | | | | | | | | | |
| Phe | UUU | 361 | 1.87 | Ser | UCU | 96 | 2.22 | Tyr | UAU | 173 | 1.72 | Cys | UGU | 108 | 1.77 |
| Phe | UUC | 26 | 0.13 | Ser | UCC | 3 | 0.07 | Tyr | UAC | 28 | 0.28 | Cys | UGC | 14 | 0.23 |
| Leu | UUA | 335 | 3.79 | Ser | UCA | 38 | 0.88 | Ter | UAA | 0 | 0.00 | Ter | UGA | 62 | 3.00 |
| Leu | UUG | 124 | 1.40 | Ser | UCG | 13 | 0.30 | Ter | UAG | 0 | 0.00 | Trp | UGG | 27 | 1.00 |
| Leu | CUU | 45 | 0.51 | Pro | CCU | 36 | 2.00 | His | CAU | 42 | 1.68 | Arg | CGU | 30 | 1.14 |
| Leu | CUC | 1 | 0.01 | Pro | CCC | 0 | 0.00 | His | CAC | 8 | 0.32 | Arg | CGC | 1 | 0.04 |
| Leu | CUA | 17 | 0.19 | Pro | CCA | 31 | 1.72 | Gln | CAA | 21 | 1.50 | Arg | CGA | 11 | 0.42 |
| Leu | CUG | 9 | 0.10 | Pro | CCG | 5 | 0.28 | Gln | CAG | 7 | 0.50 | Arg | CGG | 6 | 0.23 |
| Ile | AUU | 146 | 1.36 | Thr | ACU | 65 | 2.39 | Asn | AAU | 70 | 1.73 | Ser | AGU | 100 | 2.31 |
| Ile | AUC | 17 | 0.16 | Thr | ACC | 6 | 0.22 | Asn | AAC | 11 | 0.27 | Ser | AGC | 10 | 0.23 |
| Ile | AUA | 160 | 1.49 | Thr | ACA | 32 | 1.17 | Lys | AAA | 55 | 1.04 | Arg | AGA | 79 | 3.00 |
| Met | AUG | 89 | 1.00 | Thr | ACG | 6 | 0.22 | Lys | AAG | 51 | 0.96 | Arg | AGG | 31 | 1.18 |
| Val | GUU | 169 | 2.12 | Ala | GCU | 54 | 2.88 | Asp | GAU | 82 | 1.93 | Gly | GGU | 95 | 1.94 |
| Val | GUC | 5 | 0.06 | Ala | GCC | 1 | 0.05 | Asp | GAC | 3 | 0.07 | Gly | GGC | 14 | 0.29 |
| Val | GUA | 93 | 1.17 | Ala | GCA | 14 | 0.75 | Glu | GAA | 34 | 1.06 | Gly | GGA | 41 | 0.84 |
| Val | GUG | 52 | 0.65 | Ala | GCG | 6 | 0.32 | Glu | GAG | 30 | 0.94 | Gly | GGG | 46 | 0.94 |
| *Echinococcus equinus* | | | | | | | | | | | | | | | |
| Phe | UUU | 389 | 1.93 | Ser | UCU | 99 | 2.22 | Tyr | UAU | 200 | 1.87 | Cys | UGU | 130 | 1.83 |
| Phe | UUC | 15 | 0.07 | Ser | UCC | 3 | 0.07 | Tyr | UAC | 14 | 0.13 | Cys | UGC | 12 | 0.17 |
| Leu | UUA | 161 | 1.93 | Ser | UCA | 24 | 0.54 | Ter | UAA | 0 | 0.00 | Ter | UGA | 36 | 3.00 |
| Leu | UUG | 288 | 3.46 | Ser | UCG | 29 | 0.65 | Ter | UAG | 0 | 0.00 | Trp | UGG | 62 | 1.00 |
| Leu | CUU | 24 | 0.29 | Pro | CCU | 45 | 2.54 | His | CAU | 49 | 1.88 | Arg | CGU | 33 | 1.60 |
| Leu | CUC | 1 | 0.01 | Pro | CCC | 2 | 0.11 | His | CAC | 3 | 0.12 | Arg | CGC | 3 | 0.15 |
| Leu | CUA | 13 | 0.16 | Pro | CCA | 9 | 0.51 | Gln | CAA | 8 | 0.73 | Arg | CGA | 3 | 0.15 |
| Leu | CUG | 13 | 0.16 | Pro | CCG | 15 | 0.85 | Gln | CAG | 14 | 1.27 | Arg | CGG | 12 | 0.58 |
| Ile | AUU | 138 | 1.97 | Thr | ACU | 67 | 3.01 | Asn | AAU | 82 | 1.91 | Ser | AGU | 105 | 2.36 |
| Ile | AUC | 12 | 0.17 | Thr | ACC | 1 | 0.04 | Asn | AAC | 4 | 0.09 | Ser | AGC | 7 | 0.16 |
| Ile | AUA | 60 | 0.86 | Thr | ACA | 2 | 0.09 | Lys | AAA | 12 | 0.42 | Arg | AGA | 33 | 1.60 |
| Met | AUG | 86 | 1.00 | Thr | ACG | 19 | 0.85 | Lys | AAG | 45 | 1.58 | Arg | AGG | 40 | 1.94 |
| Val | GUU | 259 | 2.26 | Ala | GCU | 51 | 2.62 | Asp | GAU | 80 | 1.93 | Gly | GGU | 144 | 2.48 |
| Val | GUC | 7 | 0.06 | Ala | GCC | 4 | 0.21 | Asp | GAC | 3 | 0.07 | Gly | GGC | 11 | 0.19 |
| Val | GUA | 64 | 0.56 | Ala | GCA | 8 | 0.41 | Glu | GAA | 18 | 0.55 | Gly | GGA | 24 | 0.41 |
| Val | GUG | 129 | 1.12 | Ala | GCG | 15 | 0.77 | Glu | GAG | 48 | 1.45 | Gly | GGG | 53 | 0.91 |
| *Echinococcus canadensis* | | | | | | | | | | | | | | | |
| Phe | UUU | 388 | 1.91 | Ser | UCU | 107 | 2.46 | Tyr | UAU | 189 | 1.78 | Cys | UGU | 129 | 1.80 |
| Phe | UUC | 19 | 0.09 | Ser | UCC | 5 | 0.11 | Tyr | UAC | 23 | 0.22 | Cys | UGC | 14 | 0.20 |
| Leu | UUA | 156 | 1.86 | Ser | UCA | 19 | 0.44 | Ter | UAA | 0 | 0.00 | Ter | UGA | 33 | 3.00 |
| Leu | UUG | 301 | 3.60 | Ser | UCG | 25 | 0.57 | Ter | UAG | 0 | 0.00 | Trp | UGG | 65 | 1.00 |
| Leu | CUU | 24 | 0.29 | Pro | CCU | 49 | 2.72 | His | CAU | 48 | 1.85 | Arg | CGU | 36 | 1.80 |
| Leu | CUC | 1 | 0.01 | Pro | CCC | 1 | 0.06 | His | CAC | 4 | 0.15 | Arg | CGC | 0 | 0.00 |
| Leu | CUA | 6 | 0.07 | Pro | CCA | 12 | 0.67 | Gln | CAA | 9 | 0.82 | Arg | CGA | 5 | 0.25 |
| Leu | CUG | 14 | 0.17 | Pro | CCG | 10 | 0.56 | Gln | CAG | 13 | 1.18 | Arg | CGG | 11 | 0.55 |
| Ile | AUU | 147 | 2.01 | Thr | ACU | 69 | 3.03 | Asn | AAU | 76 | 1.90 | Ser | AGU | 94 | 2.16 |
| Ile | AUC | 12 | 0.16 | Thr | ACC | 1 | 0.04 | Asn | AAC | 4 | 0.10 | Ser | AGC | 11 | 0.25 |
| Ile | AUA | 60 | 0.82 | Thr | ACA | 7 | 0.31 | Lys | AAA | 12 | 0.44 | Arg | AGA | 26 | 1.30 |
| Met | AUG | 84 | 1.00 | Thr | ACG | 14 | 0.62 | Lys | AAG | 43 | 1.56 | Arg | AGG | 42 | 2.10 |
| Val | GUU | 247 | 2.25 | Ala | GCU | 54 | 2.43 | Asp | GAU | 79 | 1.93 | Gly | GGU | 159 | 2.62 |
| Val | GUC | 16 | 0.15 | Ala | GCC | 7 | 0.31 | Asp | GAC | 3 | 0.07 | Gly | GGC | 10 | 0.16 |
| Val | GUA | 50 | 0.46 | Ala | GCA | 6 | 0.27 | Glu | GAA | 21 | 0.63 | Gly | GGA | 23 | 0.38 |
| Val | GUG | 126 | 1.15 | Ala | GCG | 22 | 0.99 | Glu | GAG | 46 | 1.37 | Gly | GGG | 51 | 0.84 |
| *Dipylidium caninum* | | | | | | | | | | | | | | | |
| Phe | UUU | 471 | 1.97 | Ser | UCU | 123 | 2.70 | Tyr | UAU | 214 | 1.89 | Cys | UGU | 154 | 1.90 |
| Phe | UUC | 8 | 0.03 | Ser | UCC | 2 | 0.04 | Tyr | UAC | 12 | 0.11 | Cys | UGC | 8 | 0.10 |
| Leu | UUA | 186 | 2.31 | Ser | UCA | 24 | 0.53 | Ter | UAA | 0 | 0.00 | Ter | UGA | 37 | 3.00 |
| Leu | UUG | 240 | 2.98 | Ser | UCG | 9 | 0.20 | Ter | UAG | 0 | 0.00 | Trp | UGG | 50 | 1.00 |
| Leu | CUU | 43 | 0.53 | Pro | CCU | 53 | 2.75 | His | CAU | 51 | 1.92 | Arg | CGU | 49 | 3.34 |
| Leu | CUC | 1 | 0.01 | Pro | CCC | 2 | 0.10 | His | CAC | 2 | 0.08 | Arg | CGC | 1 | 0.07 |
| Leu | CUA | 8 | 0.10 | Pro | CCA | 14 | 0.73 | Gln | CAA | 8 | 0.80 | Arg | CGA | 2 | 0.14 |
| Leu | CUG | 5 | 0.06 | Pro | CCG | 8 | 0.42 | Gln | CAG | 12 | 1.20 | Arg | CGG | 1 | 0.07 |
| Ile | AUU | 162 | 1.91 | Thr | ACU | 62 | 2.85 | Asn | AAU | 89 | 1.96 | Ser | AGU | 112 | 2.46 |
| Ile | AUC | 5 | 0.06 | Thr | ACC | 2 | 0.09 | Asn | AAC | 2 | 0.04 | Ser | AGC | 3 | 0.07 |
| Ile | AUA | 88 | 1.04 | Thr | ACA | 19 | 0.87 | Lys | AAA | 44 | 0.96 | Arg | AGA | 21 | 1.43 |
| Met | AUG | 78 | 1.00 | Thr | ACG | 4 | 0.18 | Lys | AAG | 48 | 1.04 | Arg | AGG | 14 | 0.95 |
| Val | GUU | 261 | 2.81 | Ala | GCU | 59 | 3.37 | Asp | GAU | 85 | 1.98 | Gly | GGU | 168 | 3.38 |
| Val | GUC | 6 | 0.06 | Ala | GCC | 1 | 0.06 | Asp | GAC | 1 | 0.02 | Gly | GGC | 9 | 0.18 |
| Val | GUA | 48 | 0.52 | Ala | GCA | 8 | 0.46 | Glu | GAA | 18 | 0.63 | Gly | GGA | 10 | 0.20 |
| Val | GUG | 57 | 0.61 | Ala | GCG | 2 | 0.11 | Glu | GAG | 39 | 1.37 | Gly | GGG | 12 | 0.24 |
