# Supplementary material for: The complete mitochondrial genome of the tapeworm Cladotaenia vulturi (Cestoda: Paruterinidae): gene arrangement and phylogenetic relationships with other cestodes
Source: Parasit Vectors. 2016 Aug 31;9(1):475. doi: 10.1186/s13071-016-1769-x (PMC5006517; doi:10.1186/s13071-016-1769-x)
Supplement: Additional file 5: — Figure S3. Distance tree based on the RSCU values for 12 mt protein-coding genes of cestode species used in the phylogenetic analyses. (DOC 147 kb) [file 13071_2016_1769_MOESM5_ESM.doc]

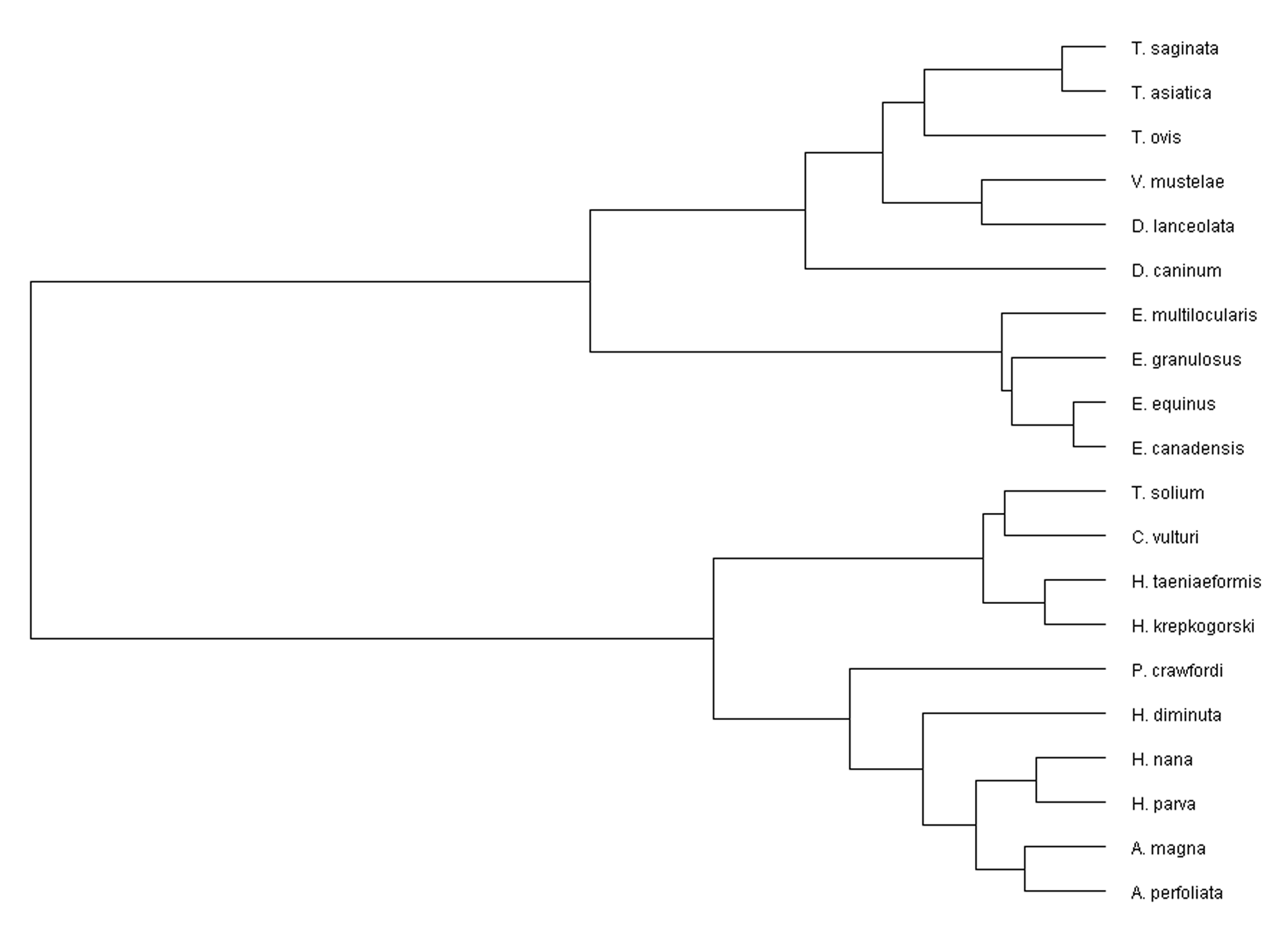


**Additional file 5: Figure S3.** Distance tree based on the RSCU values for 12 mt protein-coding genes of cestode species used in the phylogenetic analyses
